# CUT&RUNTools: a flexible pipeline for CUT&RUN processing and footprint analysis

**DOI:** 10.1186/s13059-019-1802-4

**Published:** 2019-09-09

**Authors:** Qian Zhu, Nan Liu, Stuart H. Orkin, Guo-Cheng Yuan

**Affiliations:** 1 Department of Biostatistics and Computational Biology, Dana-Farber Cancer Institute and Harvard Chan School of Public Health, Boston, MA USA; 2 Cancer and Blood Disorders Center, Dana-Farber Cancer Institute and Boston Children’s Hospital, Harvard Medical School, Boston, MA USA; 3 Howard Hughes Medical Institute, Boston, MA USA

## Abstract

We introduce CUT&RUNTools as a flexible, general pipeline for facilitating the identification of chromatin-associated protein binding and genomic footprinting analysis from antibody-targeted CUT&RUN primary cleavage data. CUT&RUNTools extracts endonuclease cut site information from sequences of short-read fragments and produces single-locus binding estimates, aggregate motif footprints, and informative visualizations to support the high-resolution mapping capability of CUT&RUN. CUT&RUNTools is available at https://bitbucket.org/qzhudfci/cutruntools/.

**Electronic supplementary material:**

The online version of this article (10.1186/s13059-019-1802-4) contains supplementary material, which is available to authorized users.

## Introduction

**Fig. 1:**
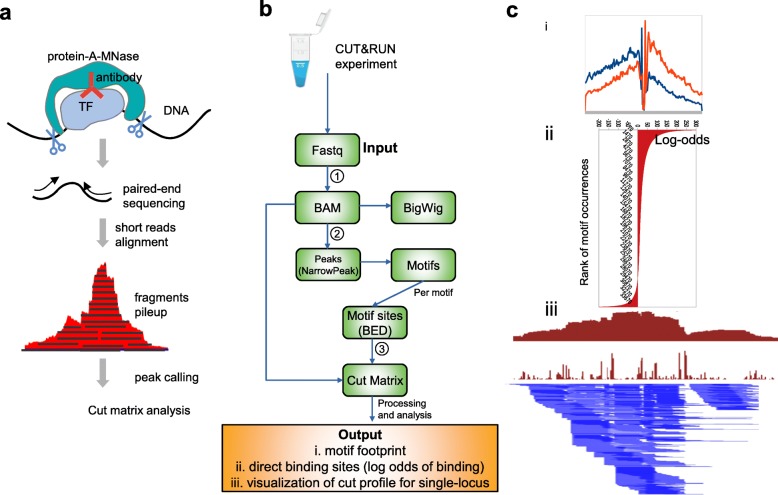
**a** Schematic of CUT&RUN. pA-MN is recruited to TF-bound antibody and cleaves around TF binding site, liberating DNA fragments for sequencing. Subsequent steps require a specially designed computational pipeline to extract maximal information from the data. **b** Overview of CUT&RUNTools. Step 1: input paired-end raw reads are aligned to the reference genome with special care for short-read trimming and alignment. Step 2: peaks are called based on fragment pileup. A fixed window around the summit of each peak is used to perform de novo motif finding. Step 3: the cut matrix is calculated for each motif of interest and used to generate the three outputs: (i) motif footprint, (ii) direct binding site identification, and (iii) visualization. **c** The output of CUT&RUNTools at the chr3:98302650-950 region as an example

Mapping the occupancy of DNA-associated proteins, including transcription factors (TFs) and histones, is central to determining cellular regulatory circuits. Conventional ChIP sequencing (ChIP-seq) relies on the cross-linking of target proteins to DNA and physical fragmentation of chromatin [1]. In practice, epitope masking and insolubility of protein complexes may interfere with the successful use of conventional ChIP-seq for some chromatin-associated proteins [2–4]. CUT&RUN is a recently described native endonuclease-based method based on the binding of an antibody to a chromatin-associated protein in situ and the recruitment of a protein A-micrococcal nuclease fusion (pA-MN) to the antibody to efficiently cleave DNA surrounding binding sites [5]. The CUT&RUN method has been successfully applied to a range of TFs in yeast [5, 6] and mammalian cells [7, 8]. The procedure achieves higher-resolution mapping of protein binding since endonuclease digestion generates shorter fragments than physical fragmentation. In our experience, existing tools to analyze such data proved inadequate due to the lack of an end-to-end computational pipeline specifically tailored to this technology. Therefore, we have developed a new pipeline, designated CUT&RUNTools, that streamlines the processing, usage, and visualization of data generated by CUT&RUN (Fig. 1a).

## Results

### Overview

**Fig. 2:**
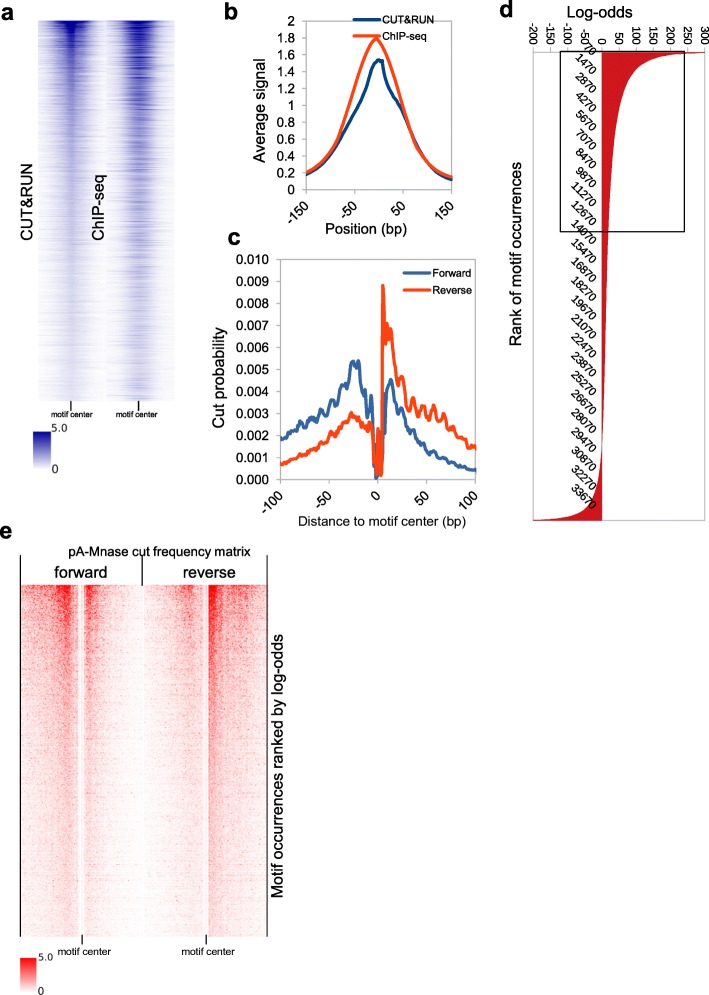
**a** GATA1 CUT&RUN and ChIP-seq comparison. GATA1 motif is scanned across CUT&RUN and ChIP-seq peaks. The signal pileup of − 150-bp to + 150-bp region surrounding each motif site is plotted. **b** CUT&RUN signal is enriched in a narrower window than ChIP-seq, consistent with a higher resolution of the CUT&RUN method. **c** CUT&RUN footprint for the HGATAA motif. Enzyme cut protection is noted in motif core and deprotection in the flanking regions. **d** The distribution of log-odds score for genome-wide HGATAA motif sites. A threshold value of 5 is used to determine direct binding sites. **e** Strand-specific cut frequency profile at individual HGTAA motif sites, illustrated as a heatmap

CUT&RUNTools takes paired-end sequencing read FASTQ files as the input and performs a set of analytical steps: trimming of adapter sequences, alignment to the reference genome, peak calling, estimation of cut matrix at single-nucleotide resolution, de novo motif searching, motif footprinting analysis, direct binding site identification, and data visualization (Fig. 1b). The outputs of the pipeline (Fig. 1c) are (1) an aggregate footprint capturing the characteristics of chromatin-associated protein binding (Fig. 1c, (i)), (2) binding log-odds values for individual motif sites informative for direct binding site identification (Fig. 1c, (ii)), and (3) visualization of a cut frequency profile at nucleotide resolution (Fig. 1c, (iii)).

Specifically, CUT&RUNTools performs sequence alignment with special attention to short-read trimming and read alignment (Fig. 1b, step 1) (the “Methods” section). Due to the predominance of short fragments (25–50 bp) generated by CUT&RUN, the typical settings in the read trimming and sequence alignment does not perform well. We introduce a two-step read trimming process to improve the quality. First, the sequencing data are processed with Trimmomatic [9], a commonly used template-based trimmer. Next, a second trimming step was included to remove any remaining adapter overhang sequences not removed due to fragment read-through. CUT&RUNTools further adjusts the default alignment settings by turning on dovetail alignment [10], designed to accept alignments for paired-end reads when there is a large degree of overlap between two mates of each pair. Together, this improved trimming and alignment procedure increased the alignment percentage from 68 to 98% compared to a setting with no trimming and alignment adjustments (Additional file 1: Table S1). With the reads aligned, CUT&RUNTools employs MACS [11] to perform peak calling based on the coverage profile, followed by de novo motif searching within the peak regions with MEME suite [12] (Fig. 1b, step 2).

### Cut matrix estimation

An important element of CUT&RUN analysis is the estimation of cut sites, which enables higher-resolution mapping of binding locations than peak calling. The cut sites derive from the two ends of individual DNA fragments generated upon cutting of chromatin by the pA-MN fusion recruited to the antibody binding sites. The regions of lower cut frequency tend to be protected due to chromatin-associated protein binding, whereas flanking regions without binding display higher cut frequencies (Additional file 2: Figure S1a). CUT&RUNTools accurately tabulates the frequency with which cleavage is observed at each base pair (the “Methods” section).

Using the cut matrix, footprinting analysis [13, 14] is then applied to identify high-resolution occupancy of sequence-specific binding factors such as TFs. To detect footprints from CUT&RUN data, CUT&RUNTools first generates an aggregated cut frequency profile based on all ± 100-bp regions extending from each peak-embedded motif site. Then, CUT&RUNTools estimates a probabilistic bimodal clustering model derived from the CENTIPEDE package [15] and assigns a binding probability score, expressed as log-odds, to each motif occurrence based on the model. This log-odds score quantifies the similarity between the cuts at each motif occurrence and the aggregate footprint pattern. By ranking the sites by the log-odds score, CUT&RUNTools generates a rank-ordered list of likely direct binding sites. Of note, this approach is only applicable to factors with distinct sequence specificity.

We illustrate the functionality of CUT&RUNTools through analysis of CUT&RUN data acquired for GATA1, a master regulator in erythroid lineage cells [16]. We performed CUT&RUN using GATA1 antibody in primary human stem/progenitor CD34+ cells after 7 days of erythroid differentiation (Fig. 2). The results were compared initially to published GATA1 ChIP-seq data for cells under the same conditions [7]. Peaks identified in CUT&RUN align very well with ChIP-seq at over 35,000 GATA1 sites across the genome (Fig. 2a). Replicate correlation was over 0.92 (Additional file 2: Figure S2). Furthermore, the pileup signal in CUT&RUN is more enriched in a narrower window in the peak center than ChIP-seq (50 bp vs. > 150 bp), reflecting higher resolution (Fig. 2b). As expected, CUT&RUNTools correctly identified the HGATAA GATA1 recognition motif de novo (*E* = 1e−200). Next, we performed GATA1 footprinting using the cut matrix generated on the HGATAA motif by CUT&RUNTools and the surrounding 150-bp regions for all 35,000 sites in the peak regions (Fig. 2c). Such footprints cannot be obtained from ChIP-seq analysis. Indication of protection at the motif core was particularly strong (Fig. 2c, e). Based on the estimated log-odds scores (Fig. 2d) (Additional file 1: Table S2), CUT&RUNTools identified 25,900 of the 35,000 motif sites as direct binding sites. Comparison with literature data validates these estimates at the locus level (Additional file 2: Figure S3), and a systematic comparison with ChIP-seq is shown in Fig. 2a. Of note, a stereotypical, center-depleted cutting pattern is identifiable not only from the average profile but also at individual motif sites.

### Application to GATA1 CUT&RUN dataset

**Fig. 3:**
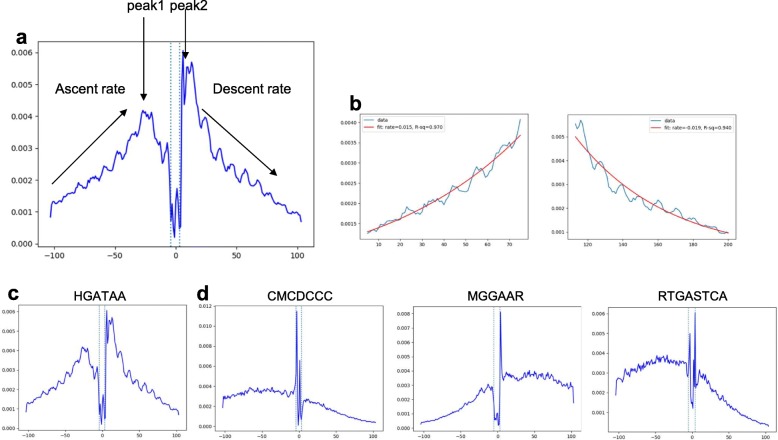
Footprint symmetry analysis reveals the primary and secondary motifs. **a** Symmetry analysis calculates the ascent and descent rates for the two sides of footprint via fitting an exponential decay curve to each part. **b** An example of exponential decay curve that is fit on real data. **c** A perfect symmetry indicates a primary motif (HGATAA). **d** A substantial difference between ascent and descent rates would indicate a motif footprint with an asymmetric footprint shape, as shown by CMCDCCC, MGGAAR, and RTGASTCA secondary motifs. Asymmetry is quantified by the footprint symmetry score (FSS)

In addition, de novo analysis of GATA1 CUT&RUN returned several additional motifs that may correspond to co-factors (Additional file 1: Table S3). These co-factor motifs (also termed secondary) can be distinguished via an asymmetrical motif footprint profile (Additional file 2: Figure S4), in contrast to the symmetrical profile of the primary HGATAA motif (Fig. 2c). We use a “footprint symmetry score” (FSS) to discriminate primary from secondary motif footprints (the “Methods” section, Fig. 3a, b) (Additional file 1: Table S4). HGATAA has the highest FSS score (Fig. 3c). Identified co-factor motifs GCCCCGCCCTC, CMCDCCC, and RTGASTCA (Fig. 3d) correspond to SP1, KLF1, and NFE2 TFs, respectively, which are known to cooperate with GATA1 [17]. Each profile displays a noticeably higher rate of descent on one side of the motif than the other (Additional file 2: Figure S4).

Importantly, de novo analysis also identified an extended motif for co-binding of GATA1 and TAL1 [18]. GATA1 forms a multiprotein complex with TAL1 along with LMO2 and Ldb1 [18, 19]. The GATA1-TAL1 complex recognizes HGATAA and a half E-box (TAL1) separated by a gap of ten nucleotides [20]. Despite the length of this motif, CUT&RUNTools displays a strong footprint for the extended motif. The high value of FSS indicates that this is a primary motif, as expected from the GATA1-TAL1 complex binding model (Additional file 2: Figure S5). The motif footprinting result is consistent between de novo and known GATA1-TAL1 motifs (Additional file 2: Figure S5). Therefore, in cases where the recognition sequence of TF is not known in advance, de novo analysis in combination with genomic footprinting should be helpful in establishing the primary motif and searching for novel co-factors.

To validate these predictions, we applied CUT&RUN to profile TAL1 and KLF1 (Additional file 2: Figure S6). Of the 19,871 predicted GATA1-TAL1 co-binding sites from GATA1 CUT&RUN, 12,841 (64.6%, Jaccard index = 0.51, *P* < 10^−5^, bootstrap test) are validated by the TAL1 CUT&RUN experiment. In the case of KLF1, 10,733 of 17,826 (60.2%, Jaccard index = 0.26, *P* < 10^−5^, bootstrap test) predicted GATA1-KLF1 co-binding sites are confirmed by KLF1 CUT&RUN analysis. These results suggest that CUT&RUNTools is useful for uncovering combinatorial regulatory modules.

### Applicability in additional CUT&RUN datasets

**Fig. 4:**
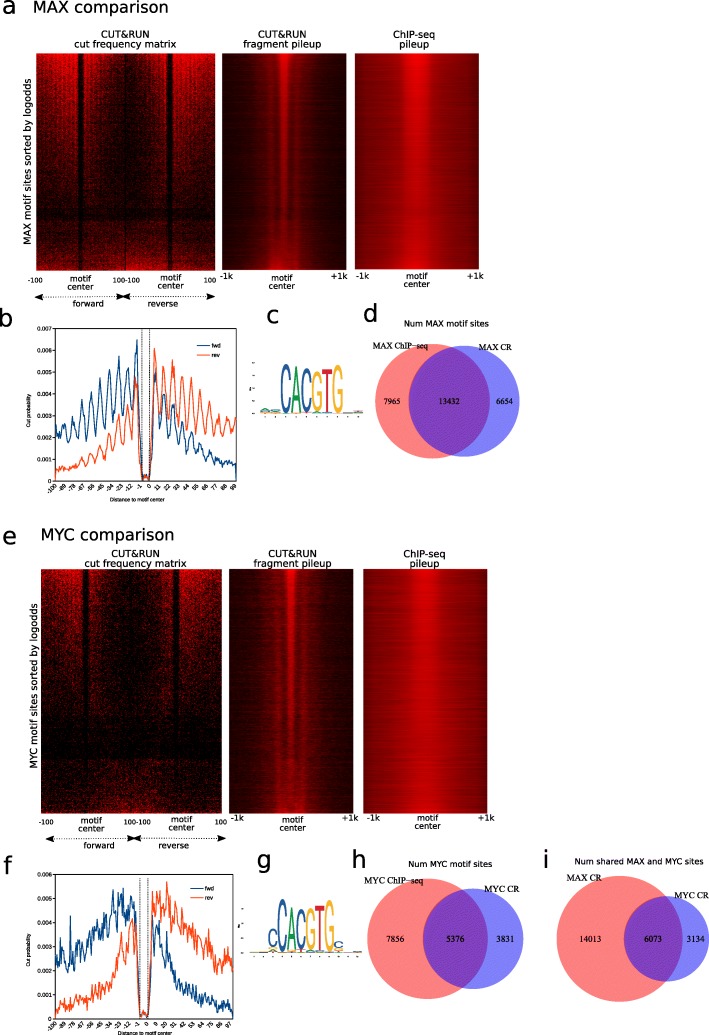
**a**–**d** MAX comparison. **e**–**h** MYC comparison. **a** MAX CUT&RUN cut frequency matrix, fragment pileup, and MAX ChIP-seq pileup (all public experiments). **b** MAX motif footprinting generated by CUT&RUNTools. **c** Enriched MAX motif used to do the motif footprinting analysis. **d** Overlap of MAX motif sites in ChIP-seq and CUT&RUN peaks. **e** MYC CUT&RUN cut frequency matrix, fragment pileup, and MYC ChIP-seq pileup (all public experiments). **f**–**h** Same as **b**–**d** except that the transcription factor is MYC. **i** Overlap of MAX and MYC motif sites in CUT&RUN experiments

To illustrate the broad applicability of our tool, we compared CUT&RUN with ChIP-seq datasets that have been generated for several factors from other labs. For example, we compared the CUT&RUN data for MAX and MYC that were performed previously [5] with the corresponding ChIP-seq experiments from K562 cells in the ENCODE database. Analyses of MAX and MYC experiments using CUT&RUNTools identified 23,153 and 6996 peaks, respectively, which are well enriched in the respective TF motifs—9207 motif sites for MYC and 20,086 motif sites for MAX have been identified, see Fig. 4. As expected, CUT&RUN has improved resolution over ChIP-seq (Fig. 4a, e). We found 6073 out of 9207 instances where MYC and MAX share binding sites or have very close binding locations (< 10 bp) (Fig. 4i), confirming the known dimerization between these two factors. Interestingly, MAX CUT&RUN revealed a 10-bp periodic cut pattern in its summary footprint profile (Fig. 4b). A previous study has found via DNase I footprinting of MAX the enhanced cleavage in nucleosomal DNA separated by areas of increased protection at 10-bp periodicity [21], validating our footprint profile. Like MAX, we believe that CUT&RUN is useful for investigating the nucleosomal binding of transcription factors, in addition to binding at the nucleosome-free regions. It should be noted that to locate nucleosomal binding, the > 120-bp fraction should also be used in CUT&RUNTools as we have done for MYC and MAX.

### Comparison with existing software packages

Several tools are available for estimating cut matrices from ATAC-seq and DNase-seq data [22, 23]. However, the direct application of such tools to analyze CUT&RUN data often leads to incorrect estimates due to the differences in the experimental protocol (Additional file 2: Figure S7, S8). One reason is that the two ends of each mate of paired reads both do not indicate the ends of a fragment (Additional file 2: Figure S9), making the accounting of cut positions challenging. Another important difference is that Tn5 transposase in ATAC-seq leaves a 4-bp overhang in sequenced fragments [24], whereas pA-MN enzyme in CUT&RUN cleaves surrounding the location of binding sites with no overhang. Specific adjustments are thus required and have been made in the enumeration of the cut matrix to take into account this feature of CUT&RUN (the “Methods” section). Recognizing these differences, we provide an option to tune the cut site offset to make CUT&RUNTools applicable to both CUT&RUN and ATAC-seq footprinting analyses (Additional file 2: Figure S10) and in doing so allow flexibility of experiment type.

**Fig. 5:**
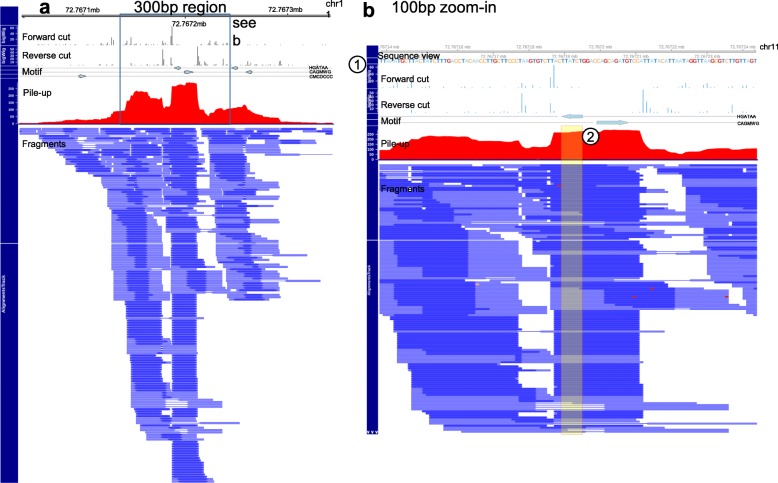
**a** CUT&RUNTools visualization of an example region chr11:72767100-72767300. The top two tracks show the strand-specific cut frequency profiles. The third track is the signal pileup plot. The fourth track is the fragment plot, showing the location and start and end positions of each DNA fragment. Forward cut frequency refers to the end of R1 mate and reverse refers to the end of R2 mate, where the designation of R1 and R2 is based on whether the mate alignment contains the motif or the motif’s reverse complement. **b** A zoom-in view of the same region as **a**. The view contains an additional sequence view (1) and highlighting of HGATAA motif (2)

Finally, CUT&RUNTools includes several quality control metrics, including fragment size distribution, read duplication rate, library size, adapter content percentage, and alignment percentage to assist users in quality control evaluation of CUT&RUN experiments (the “Methods” section). By further using the number of peaks and the enrichment of the expected motif, users can evaluate the overall success of experiments and validate a given antibody. Additionally, CUT&RUNTools generates publication-quality visualizations to aid biologists in interpreting cleavage data and to substantiate evidence of binding (Fig. 5). The cut frequency track, for example, displays the number of cuts at each nucleotide position within a specified genomic range. A broad-level visualization (300 bp) (Fig. 5a) highlights the location of the motif and other footprints within the region. At 100-bp resolution (Fig. 5b), a genomic sequence view is enabled and the exact locations of cleavage can be seen. These visualizations can be executed simply through user-friendly commands. CUT&RUNTools supports SLURM-based [25] cluster environment and permits simple specification of inputs/outputs, tools, and resource-related parameters through a JSON-formatted configuration file. A detailed usage manual is provided online.

Quality control measures such as alignment rate and fragment duplication rate may be used to evaluate CUT&RUN, but we note that due to the differences in the mappability and sequence composition of the antibody-bound DNA, some factors inherently have a low complexity of the binding regions and an increased fragment duplication rate. Users, therefore, need to make judgment calls, for example, whether or not to remove duplicates, on a factor-dependent basis. We also advise an interpretation of the data that is aided by motif and replicate analysis.

## Methods

### CUT&RUN experiments

CUT&RUN experiments were carried out following the nuclei isolation version of the protocol as described [5, 7]. Nuclei from 2 × 10^6^ cells were isolated with NE buffer that consisted of 20 mM HEPES-KOH pH 7.9, 10 mM KCl, 0.5 mM spermidine, 0.1% Triton X-100, 20% glycerol, and 1× protease inhibitor cocktails. The nuclei were captured with BioMagPlus Concanavalin A and incubated with 2 μg primary antibody (α-GATA1, ab11852, Abcam) in 200 μL wash buffer (20 mM HEPES-NaOH pH 7.5, 150 mM NaCl, 0.5 mM spermidine, 0.1% BSA, and 1× protease inhibitor cocktails) for 2 h. Then, unbound antibody was washed away with 400 μL wash buffer twice. Then pA-MN was added at 1:1000 ratio to 200 μL wash buffer and incubated for 1 h. The nuclei were washed again and resuspended in 150 μL wash buffer. CaCl_2_ was next added at a final concentration of 2 mM to activate the enzyme. The reaction was carried out at 0 °C and stopped by 150 μL of 2X STOP buffer (200 mM NaCl, 20 mM EDTA, 50 μg/mL RNase A, and 40 μg/mL glycogen). Protein-DNA complex was released by centrifugation and digested by proteinase K at 50 °C overnight, followed by DNA precipitation by ethanol. The pellet was washed with 70% ethanol and dissolved in 25 μL 0.1× TE (1 mM Tris-HCl pH 8.0, 0.1 mM EDTA). Antibody used for TAL1 and KLF1 CUT&RUN were ab155195 (Abcam) and HPA051850 (Sigma), respectively.

### CUT&RUN library preparation and sequencing

The NEBNext Ultra II DNA Library Prep Kit was used with modifications described previously [7] which aims to preserve short DNA fragments (30–80 bp). Briefly, 6 ng of CUT&RUN DNA were treated with endprep module at 20 °C for 30 min and 50 °C for 1 h to reduce the melting of short DNA. Ligation was performed by adding 5 pmol of NEB adapter and ligation mix and incubated at 20 °C for 15 min. To clean up the reaction, add 1.75× volume of Agencourt AMPure XP beads (Beckman Coulter) to capture short ligation products. PCR amplification was performed for 12 cycles. The resulting libraries were purified with 1.2× volume of AMPure beads then analyzed and quantified by Qubit and Tapestation. The detailed step-by-step protocol can be found at protocol.io (10.17504/protocols.io.wvgfe3w). Libraries with different indexes were pooled, and Illumina paired-end sequencing was performed using Nextseq 500 platform with NextSeq 500/550 High Output Kit v2 (75 cycles) (2 × 42 bp, 6-bp index).

### Detailed implementations

Broadly, CUT&RUNTools consists of trimming, alignment, peak calling, motif finding, cut matrix generation, and motif footprinting steps. The pipeline incorporates specific changes to some of the steps to accommodate the short-read and short fragment characteristics of CUT&RUN. Its cut matrix generation ensures an accurate accounting of cut positions for footprint analyses. These steps are described below.

#### Raw read trimming and alignment

Short fragments are frequently encountered in CUT&RUN experiments due to the fine cutting by pA-MN enzyme. As a result, it is common to expect both mates of DNA fragment to overlap. When the fragment is shorter than the length of a read, then we can expect that adapter run-through will occur. It is thus critical to remove adapter sequences at the end of the reads. To deal with the issues caused by the alignment of short fragments, we made two important modifications to the typical adapter trimming and alignment protocol:

1. An initial trimming was first performed with Trimmomatic [9], with settings optimized to detect adapter contamination in short-read sequences. Trimmomatic is a template-based trimmer. However, reads containing 6 bp, or less, of adapters are not trimmed. Therefore, a separate tool Kseq was developed to trim up to 6-bp adapters from the 3′ end of each read that was not effectively processed by Trimmomatic. Note that this trimming does not affect the cut site calculation, which counts only the 5′ end of sequences. After trimming, a minimum read length of 25 bp was imposed, as reads smaller than this were hard to align accurately.
2. Dovetail alignment policy. Bowtie2 [10] aligns each mate of a pair separately and then discards any pairs that have been aligned inconsistently. Dovetail refers to the situation when mates extend past each other. In the default setting, these alignments are discarded. Dovetail is unusual but encountered in CUT&RUN experiments. The --dove-tail setting [10] was enabled to flag this situation as normal or “concordant” instead of elimination of such reads.

#### Peak calling and motif finding

After alignment, fragments were divided into ≤ 120-bp and > 120-bp fractions. For the rest of the analyses, we used the ≤ 120-bp fraction which is likely to contain TF binding sites [5]. Then, MACS2 was applied with the default narrowPeak setting [11]. Afterward, sequences within 100 bp from the summit of each peak were obtained, and any sequences containing a substantial amount of repeats (as reported by RepeatMask) were removed. These remaining sequences were next used to perform de novo motif searching using MEME [12]. The top 20 motifs were saved for subsequent analyses. FIMO (part of MEME suite [12]) was applied to enumerate all motif sites in the peak regions.

Like other techniques, some fraction of sequenced read pairs appears as duplicates (i.e., with identical start and end positions between duplicates). However, it is argued that nuclease cleavage of chromatin by its stereotypical nature is influenced by conformation of chromatin and/or nuclease bias [26], and shorter DNA fragments also increased the likelihood of identical reads that originated from different cells [27]. Thus, removing duplicates from CUT&RUN experiments should be dealt with caution if the library complexity is not too low (due to extremely low input and/or high PCR cycle numbers). Thus, the default action in CUT&RUNTools is to retain duplicate reads, and users can choose to remove duplicates at their own discretion. We recommend users to be aware of the low complexity of libraries with high duplication rates, as these may indicate a poor quality preparation. Users may repeat peak calling analysis on both duplicate and duplicate-removed instances. By comparing the peak number, motif enrichment, enrichment of expected motifs, and other quality metrics, users may decide whether it makes sense to use the duplicate version for subsequent analysis.

#### Cut matrix generation

For any motif of interest, its corresponding cut matrix was generated as follows. The rows of the cut matrix are the motif sites. The columns are the individual nucleotides in the − 100-bp motif and + 100-bp regions. Cut matrix requires all motif sites to be in a consistent orientation. That is, if the motif occurrence is located on the minus strand in the reference genome, all the cut frequencies in that motif site are flipped, so that − 100-bp position from the old profile becomes the + 100-bp position in the new profile. By convention, a value at *i*th nucleotide means the cut is situated just before *i*th nucleotide. The cut matrix tabulates the frequency of fragments ending in each nucleotide.

To compute strand-specific cut matrix, the ends of DNA fragments that overlap with the motif were assigned to forward and reverse strand cut matrices as follows. For each fragment, define R1 and R2 as two mates. The ends of the fragment are the start of R1 (*s*_1_) and the end of R2 (*e*_2_). If a given motif occurrence appears on the positive strand of the reference genome, then *s*_1_ belongs to the “forward” strand cut and *e*_2_ belongs to the “reverse” strand cut. Otherwise, if the motif occurrence is on the negative strand, then *s*_1_ belongs to the “reverse” strand cut and *e*_2_ belongs to the “forward” strand cut. Likewise, tabulation was repeated for all paired reads and for all motif occurrences, each time separately for each strand.

#### Motif footprinting analysis

A motif footprint is a plot that shows the enzyme cleavages around the motif region, presumably due to the protection of TF-bound DNA. It is typically characterized by a low-cut frequency (or low posterior probability of cut) in the motif core and a high-cut frequency in the motif flanking regions. Prior to footprint analysis, blacklisted regions were excluded from the peak list. Any chromosome M peaks were also excluded. Next, CENTIPEDE [15] was applied to fit a probabilistic bimodal clustering model on the strand-specific cut matrix data which has aligned and centered all motif-containing regions. CENTIPEDE was run with default settings and specifying the length of the motif.

#### Footprint symmetry analysis for identification of primary and secondary motifs

CUT&RUNTools has built in a feature to determine whether a motif footprint is primary or secondary, based on a “footprint symmetry score” (FSS) defined as follows. The footprint profile is first divided in the middle into two halves, and to capture shape information, each half is fitted by an exponential decay curve (of the form *A*_left_ exp (*B*_left_ × *x*) and *A*_right_ exp (*B*_right_) × *x*, respectively) (Fig. 3). The parameter *B*_left_ (and *B*_right_) reflects the ascent rate for the left arm (and the “descent rate” for the right arm). The goodness of fit is quantified using the *R*^2^ statistic, represented by *R*^2^_left_ and *R*^2^_right_. The FSS score is defined as *B*_left_ × *R*^2^_left_ + − 1 × *B*_right_ × *R*^2^_right_. Intuitively, the FSS score measures the rate of increase of cut probabilities in the footprint plot, as the position approaches the motif. This rate should match the respective rate of decrease of cut probabilities as the position is further away from center. A FSS score of > 0.3 and a small difference between *B*_left_ and − 1 × *B*_right_ indicate symmetry of motif footprint. Such a motif is designated primary.

#### Determining direct binding sites

The criteria we used for direct binding sites were as follows: (1) the site must contain a primary motif, (2) the site must fall within a CUT&RUN peak, and (3) the site must have a high binding log-odds, which assesses the compatibility of the cut frequencies at the site with the binding model. Binding log-odds, estimated by CENTIPEDE, is defined as log(*p*/(1 − *p*)) where *p* is the overall posterior probability of binding at each site. The posterior probability for bound case (*p*) is estimated from a multinomial distribution and uses information from the spatial distribution of reads around the motif:

$$ p=R!\prod \limits_{s=1}^S\left(\frac{\lambda_s^{X_s}}{X_s!}\right) $$

where *R* is the total of reads in the region (modeled with a negative binomial distribution), *s* is a position index in the motif, *λ*_*s*_ is the per position posterior probability of cutting, and *X*_*s*_ is the per position number of reads. In the null case (no binding), *λ*_*s*_ is equal to 1/*S* or uniform. Because posterior log-odds log(*p*/(1 − *p*)) is a likelihood ratio, its estimation can use a shorter derived form for simpler numerical computation (see CENTIPEDE [15]). Running CENTIPEDE on a primary motif would satisfy the first two of three criteria already, since footprinting is performed on CUT&RUN peak regions only. Based on the CENTIPEDE result, we set a stringent cutoff of log-odds > 5 to obtain direct binding sites for the motif.

#### Implementation

CUT&RUNTools was implemented using Python, R, and BASH scripts. Visualizations of motif footprints were implemented using matplotlib library in Python. Visualization of single-locus cut profile was implemented using the Gviz R package [28]. Integration of next-generation sequencing tools was achieved using Python and BASH scripts. Configuration of pipeline, including inputs/outputs and prerequisite paths, is specified by a JSON-formatted file. CUT&RUNTools works under the SLURM [25] job submission environment. A usage manual is provided online at the repository link: https://bitbucket.org/qzhudfci/cutruntools.

### Comparison with existing tools

There are two currently available tools for enumerating cut matrices from enzyme cleavage data. One is Atactk, designed for ATAC-seq data, and the other is CENTIPEDE.tutorial, targeted towards DNase-seq. These tools were each applied to CUT&RUN data for the purpose of showing the advantage of CUT&RUNTools. Make-cut-matrix tool from the Atactk package [22] v0.1.5 was downloaded from https://github.com/ParkerLab/atactk, and the CENTIPEDE.tutorial package v1.0 was downloaded from https://github.com/slowkow/CENTIPEDE.tutorial. Make-cut-matrix was run with default settings on GATA1 CUT&RUN data, using HGATAA as the motif. The centipede_data() function of CENTIPEDE.tutorial package was used to generate cut matrix with default parameters. To evaluate the quality of the cut matrix generated by these tools, CENTIPEDE motif footprinting was performed on the generated cut matrices, and the quality of the motif footprint plot was inspected for differences. Two loci were selected to more specifically compare the cut frequency profile estimated by these tools and CUT&RUNTools and illustrate their differences.

To make sure that the cut matrix is accurately estimated for CUT&RUN data, CUT&RUNTools adapts the following changes starting with the make-cut-matrix implementation. Adjustments are written in the form of a “patch,” which is available in the pipeline. First, the default setting of 4-bp cut site offset was removed as it was usually required for ATAC-seq data (due to Tn5 transposase imposing a 4-bp overhang on the sequences [24]). CUT&RUN cuts approximately at the TF binding site, so no cut site offset is required (offset = 0). Second, the position of the reverse strand cut site is noted to be shifted by 1 bp even after setting cut site offset to be 0 (Additional file 2: Figure S11a). This shift has been a remnant feature of ATAC-seq where forward strand has a cut offset of 4 bp while the reverse strand has a cut offset of 5 bp. So, an adjustment of the cut position has been further made to correct this behavior (Additional file 2: Figure S11b). With both of these changes adapted, the cut matrix was independently verified with the fragment end positions produced by bamtobed tool from BEDTools [29] to ensure its accuracy.

### Quality control metrics

CUT&RUNTools reports a number of metrics to evaluate the quality of a CUT&RUN dataset, including fragment size distribution, adapter content percentage, library size, read duplication rate, alignment percentage, number of peaks, and enrichment of expected motif. The fragment size is measured by the start and end positions of a pair of reads in paired-end sequencing. Since the experimental protocol enriches short fragments, it is a routine to ensure that the fragment size is within the expected range (e.g., ≤ 120 bp). The quality of sequence reads is evaluated by the adapter content percentage, which is the percentage of reads retained after the read trimming step. For a good-quality dataset, the number of reads removed by trimming should be less than 10–15%, mostly corresponding to short fragments. A substantially higher number may indicate technical problems such as self-ligation. The library size, which is defined the number of reads in the sample library, should be at minimum 10 million and ideally at least ~ 15–20 million. The read duplication rate is defined as the fraction of paired reads that have identical starts for the first mate and ends for the second mate. A good-quality data should typically have a low read duplication rate (10–15%), although the rate may be higher for factors with an affinity for low-complexity regions. The alignment percentage is computed as the percentage of reads that can be mapped concordantly to the reference genome. For a good dataset, the alignment percentage should be high (e.g., > 90%). CUT&RUNTools detects peaks by applying MACS2 [11] after filtering out a number of uninteresting regions (including RepeatMasked regions, chromosome M, and any blacklisted regions). In case there is prior knowledge regarding the expected number of peaks, this may also serve as a guide to evaluate the quality of the data. For transcription factors with known sequence specificity, the enrichment of the expected motif should be high at the detected peaks. As there is no single score that captures the overall quality, the users are encouraged to make their own judgment call by considering the collective information.

### Installation and usage

Installation instructions are provided at https://bitbucket.org/qzhudfci/cutruntools/src/default/. To use the pipeline, users first create a new job which entails modifying the provided JSON configuration file with information about the sample fastq file path, output path, SLURM resource requirements, and various settings. Then, execute ./create_scripts.py config.json to create a working directory and a set of tailored SLURM submission scripts. Finally, to start the analysis for a sample of interest, users simply execute ./integrated.all.steps.sh GATA1_R1_001.fastq.gz. This script will perform the entire analysis pipeline via a 1-command interface. Options are also available for running the steps of the pipeline individually (see the manual on the website for details).

### Public dataset analysis

In the GATA1 study, GATA1, TAL1, KLF1, and NFE2 ChIP-seq experiments were downloaded from GEO. In the MAX and MYC example, public CUT&RUN samples were downloaded from GEO and compared against ChIP-seq experiments from the ENCODE consortium, see the “Availability of data and materials” section for accession IDs. ChIP-seq raw reads were trimmed, aligned, and subjected to peak calling following standard MACS2 narrow peak settings (-q 0.01 -B –SPMR) [9–11]. CUT&RUN datasets were processed using CUT&RUNTools using the default trimming and alignment settings. For MYC and MAX CUT&RUN, fragments of all sizes were kept so as to capture both free DNA and nucleosomal DNA binding. For TAL1 and KLF1 CUT&RUN, fragments of sizes ≤ 120 bp were selected for downstream analysis. Then, fragments in BAM files were subject to peak calling with MACS2 (default narrow peak settings). To compare with ChIP-seq, we subset the ChIP-seq experiment to most significant peaks to the extent that the resulting peak number is similar to the total peak number in the corresponding CUT&RUN experiment. Where the peak coverage is higher in CUT&RUN than ChIP-seq, subsetting was done instead in CUT&RUN. Then, we performed motif scanning using FIMO [12] to locate peaks containing enriched motif for the factor. The motif instances within the peaks were next overlapped between CUT&RUN and ChIP-seq, and a Venn diagram was drawn [30]. The significance of the overlap was computed using the Jaccard R package using the bootstrap method with bootstrap iterations set to 100,000. Motif scanning and footprinting analyses used the following reference motifs from JASPAR database [31]: MA0140.2 (GATA1.TAL1), MA0493.1 (KLF1), MA0841.1 (NFE2), MA0035.2 (GATA1), MA0058.3 (MAX), and MA0147.3 (MYC). FIMO motif scanning *P* value of 0.0005 was used for all motifs, except MA0035.2 that used *P* = 0.001 due to the motif’s short length.

## Discussion

In summary, CUT&RUNTools provides a means of directly detecting TF binding through assessment of the protection of TF-bound DNA from enzyme cleavages and should enable biologists to realize advantages provided by CUT&RUN. Thus, CUT&RUNTools represents a valuable enabling tool for genomic biologists to analyze and interpret CUT&RUN data and extend insights into the regulatory mechanisms.

## Additional files

Additional file 1: Supplementary Tables S1 - S4. (PDF 46 kb)

Additional file 2: Supplementary Figures S1 - S11. (PDF 4187 kb)

Additional file 3: Review history. (DOCX 16 kb)

## Data Availability

CUT&RUNTools is released under the open-source GNU General Public License v2.0 and is available at https://bitbucket.org/qzhudfci/cutruntools/src/default/. A copy of the source code has been deposited in Zenodo (doi: 10.5281/zenodo.3374112) [32]. Raw sequencing reads for the CUT&RUN experiments have been deposited at GEO (GSE136251 [33]). We also referenced public datasets in this paper. In the GATA1 study, GATA1 ChIP-seq (GSM2452102), TAL1 ChIP-seq (GSM1067277), KLF1 ChIP-seq (GSM1067275), and NFE2 ChIP-seq (GSM1067276) from GEO series GSE93372 [34] and GSE43625 [35] were used. In the MAX and MYC example, public CUT&RUN samples GSM2433145 and GSM2433146 from GSE84474 [5] were downloaded and compared against the ChIP-seq experiments from the ENCODE consortium (ENCFF713RWU and ENCFF172YQZ) [36].

## References

[1] Solomon MJ, Varshavsky A (1985). Formaldehyde-mediated DNA-protein crosslinking: a probe for in vivo chromatin structures. Proc Natl Acad Sci.

[2] Baranello L, Kouzine F, Sanford S, Levens D (2016). ChIP bias as a function of cross-linking time. Chromosom Res.

[3] Meyer CA, Liu XS (2014). Identifying and mitigating bias in next-generation sequencing methods for chromatin biology. Nat Rev Genet.

[4] Teytelman L, Thurtle DM, Rine J, van Oudenaarden A (2013). Highly expressed loci are vulnerable to misleading ChIP localization of multiple unrelated proteins. Proc Natl Acad Sci.

[5] Skene PJ, Henikoff S. An efficient targeted nuclease strategy for high-resolution mapping of DNA binding sites. Elife. 2016;6:1–35. 10.7554/eLife.21856 PMC5310842 28079019

[6] Warfield L, Ramachandran S, Baptista T, Devys D, Tora L, Hahn S (2017). Transcription of nearly all yeast RNA polymerase II-transcribed genes is dependent on transcription factor TFIID. Mol Cell.

[7] Liu N, Hargreaves VV, Zhu Q, Kurland JV, Hong J, Kim W (2018). Direct promoter repression by BCL11A controls the fetal to adult hemoglobin switch. Cell..

[8] Roth TL, Puig-Saus C, Yu R, Shifrut E, Carnevale J, Li PJ (2018). Reprogramming human T cell function and specificity with non-viral genome targeting. Nature..

[9] Bolger AM, Lohse M, Usadel B (2014). Trimmomatic: a flexible trimmer for Illumina sequence data. Bioinformatics..

[10] Ben L, Steven S (2013). Fast gapped-read alignment with Bowtie 2. Nat Methods.

[11] Zhang Y, Liu T, Meyer CA, Eeckhoute J, Johnson DS, Bernstein BE, et al. Model-based analysis of ChIP-Seq (MACS). Genome bio. 2015:1–9. 10.1186/gb-2008-9-9-r137 PMC2592715 18798982

[12] Machanick P, Bailey TL (2011). MEME-ChIP: motif analysis of large DNA datasets. Bioinformatics..

[13] Neph Shane, Vierstra Jeff, Stergachis Andrew B., Reynolds Alex P., Haugen Eric, Vernot Benjamin, Thurman Robert E., John Sam, Sandstrom Richard, Johnson Audra K., Maurano Matthew T., Humbert Richard, Rynes Eric, Wang Hao, Vong Shinny, Lee Kristen, Bates Daniel, Diegel Morgan, Roach Vaughn, Dunn Douglas, Neri Jun, Schafer Anthony, Hansen R. Scott, Kutyavin Tanya, Giste Erika, Weaver Molly, Canfield Theresa, Sabo Peter, Zhang Miaohua, Balasundaram Gayathri, Byron Rachel, MacCoss Michael J., Akey Joshua M., Bender M. A., Groudine Mark, Kaul Rajinder, Stamatoyannopoulos John A. (2012). An expansive human regulatory lexicon encoded in transcription factor footprints. Nature.

[14] Hesselberth JR, Chen X, Zhang Z, Sabo PJ, Sandstrom R, Reynolds AP (2009). Global mapping of protein-DNA interactions in vivo by digital genomic footprinting. Nat Methods.

[15] Pique-Regi R, Degner JF, Pai AA, Gaffney DJ, Gilad Y (2011). Accurate inference of transcription factor binding from DNA sequence and chromatin accessibility data. Genome Res.

[16] Pevny L, Simon MC, Robertson E, Klein WH, Tsai SF, D’Agati V (1991). Erythroid differentiation in chimaeric mice blocked by a targeted mutation in the gene for transcription factor GATA-1. Nature..

[17] Hasegawa A, Shimizu R. GATA1 activity governed by configurations of cis-acting elements. Front Oncol. 2017;6:1-7. 10.3389/fonc.2016.00269 PMC5220053 28119852

[18] Wilkinson-White L, Gamsjaeger R, Dastmalchi S, Wienert B, Stokes PH, Crossley M (2011). Structural basis of simultaneous recruitment of the transcriptional regulators LMO2 and FOG1/ZFPM1 by the transcription factor GATA1. Proc Natl Acad Sci.

[19] Wadman IA, Osada H, Grütz GG, Agulnick AD, Westphal H, Forster A (1997). The LIM-only protein Lmo2 is a bridging molecule assembling an erythroid, DNA-binding complex which includes the TAL1, E47, GATA-1 and Ldb1/NLI proteins. EMBO J.

[20] Kassouf MT, Hughes JR, Taylor S, McGowan SJ, Soneji S, Green AL (2010). Genome-wide identification of TAL1’s functional targets: insights into its mechanisms of action in primary erythroid cells. Genome Res.

[21] Wechsler DS, Papoulas O, Dang CV, Kingston RE (1994). Differential binding of c-Myc and max to nucleosomal DNA. Mol Cell Biol.

[22] Varshney A, Scott LJ, Welch RP, Erdos MR, Chines PS, Narisu N (2017). Genetic regulatory signatures underlying islet gene expression and type 2 diabetes. Proc Natl Acad Sci.

[23] Piper Jason, Elze Markus C., Cauchy Pierre, Cockerill Peter N., Bonifer Constanze, Ott Sascha (2013). Wellington: a novel method for the accurate identification of digital genomic footprints from DNase-seq data. Nucleic Acids Research.

[24] Buenrostro JD, Giresi PG, Zaba LC, Chang HY, Greenleaf WJ (2013). Transposition of native chromatin for fast and sensitive epigenomic profiling of open chromatin, DNA-binding proteins and nucleosome position. Nat Methods.

[25] Yoo AB, Jette MA, Grondona M (2003). SLURM: simple linux utility for resource management.

[26] Vierstra J, Stamatoyannopoulos JA (2016). Genomic footprinting. Nat Methods.

[27] Fu Y, Wu PH, Beane T, Zamore PD, Weng Z. Elimination of PCR duplicates in RNA-seq and small RNA-seq using unique molecular identifiers. BMC Genomics. 2018;19:1-14. 10.1186/s12864-018-4933-1 PMC6044086 30001700

[28] Hahne F, Ivanek R (2016). Visualizing genomic data using Gviz and bioconductor. Methods Mol Biol.

[29] Quinlan AR (2014). BEDTools: the Swiss-Army tool for genome feature analysis. Curr Protoc Bioinforma.

[30] Chen H, Boutros PC. VennDiagram: a package for the generation of highly-customizable Venn and Euler diagrams in R. BMC Bioinformatics. 2011;12:1-7. 10.1186/1471-2105-12-35 PMC3041657 21269502

[31] Khan A, Fornes O, Stigliani A, Gheorghe M, Castro-Mondragon JA, van der Lee R, et al. JASPAR 2018: update of the open-access database of transcription factor binding profiles and its web framework. Nucleic Acids Res. 2017; Available from: http://academic.oup.com/nar/article/doi/10.1093/nar/gkx1126/4621338. 10.1093/nar/gkx1126 PMC5753243 29140473

[32] Zhu Q. CUT&RUNTools. Bitbucket. 2019;doi:10.5281/zenodo.3374112. Available from: https://bitbucket.org/qzhudfci/cutruntools/

[33] Zhu Q, Liu N, Yuan G, Orkin S. CUT&RUNTools: a flexible pipeline for CUT&RUN processing and footprint analysis. Raw sequencing reads. Gene Expression Omnibus. 2019; https://www.ncbi.nlm.nih.gov/geo/query/acc.cgi?acc=GSE136251. Accessed 24 Aug 2019. 10.1186/s13059-019-1802-4 PMC6734249 31500663

[34] Canver MC, Wu Y, Stern EN, Needleman AJ, Chen DD, Das PP (2017). Variant-aware saturating mutagenesis using multiple Cas9 nucleases identifies regulatory elements at trait-associated loci. Nat Genet.

[35] Su MY, Bogardus H, Schulz VP, Gallagher PG, Steiner LA, Mishra T (2013). Identification of biologically relevant enhancers in human erythroid cells. J Biol Chem.

[36] Dunham I, Kundaje A, Aldred SF, Collins PJ, Davis CA, Doyle F (2012). An integrated encyclopedia of DNA elements in the human genome. Nature.

