# Supplementary material for: CUT&RUNTools: a flexible pipeline for CUT&RUN processing and footprint analysis
Source: Genome Biol. 2019 Sep 9;20:192. doi: 10.1186/s13059-019-1802-4 (PMC6734249; doi:10.1186/s13059-019-1802-4)
Supplement: Supplementary file 1 — Supplementary Tables S1 - S4. (PDF 46 kb) [file 13059_2019_1802_MOESM1_ESM.pdf]

Tables S1-S4

Table S1: improvement of reads utilization by custom trimming, dovetail alignment

	Setting	Number of reads	Aligned concordantly 1 or more times		Total	Alignment rate
			1 time	>1 times		
CUT&RUNTools	default	28787551	13070514	15380579	28451093	0.9883
	no dovetail	28787551	12804553	15075315	27879868	0.9685
	no trimming	31515085	11288822	12932597	24221419	0.7686
	no trimming and no dovetail	31515085	9003955	11327689	20331644	0.6451

Table S2: Binding log-odds* for HGATAA motif from GATA1 CUT&RUN

Chromosome	Start	End	Motif	-	Orientation	Log-odds
chr1	206084286	206084292	1-HGATAA-1-chr1	31.5	-	539.94350133
chr3	128775880	128775886	1-HGATAA-1-chr3	34.5	+	539.53606379
chr19	51161579	51161585	1-HGATAA-2-chr19	34.5	+	538.99925407
chr15	66097635	66097641	1-HGATAA-3-chr15	31.5	+	538.70591937
chr15	66097669	66097675	1-HGATAA-1-chr15	34.5	-	526.8836613
chr21	46831592	46831598	1-HGATAA-3-chr21	30	-	511.70845847
chr9	135674727	135674733	1-HGATAA-1-chr9	34.5	+	488.71658446
chr14	103844534	103844540	1-HGATAA-1-chr14	34.5	-	483.96802505
chr4	154001237	154001243	1-HGATAA-1-chr4	34.5	-	445.57355961
chr17	40781892	40781898	1-HGATAA-1-chr17	34.5	+	421.86582392
chr8	21721189	21721195	1-HGATAA-2-chr8	31.5	+	407.023727
chr20	4173122	4173128	1-HGATAA-2-chr20	31.5	+	404.41183257
chr15	31270710	31270716	1-HGATAA-2-chr15	34.5	+	402.57658532
chr4	144924560	144924566	1-HGATAA-2-chr4	31.5	-	401.80075706
chr12	122309495	122309501	1-HGATAA-2-chr12	31.5	-	396.81915852
chr17	31224658	31224664	1-HGATAA-1-chr17	34.5	+	386.97191045
chr4	144806254	144806260	1-HGATAA-2-chr4	31.5	-	377.69801447
chr17	17155717	17155723	1-HGATAA-2-chr17	34.5	+	369.99541714
chr3	42878291	42878297	1-HGATAA-2-chr3	34.5	+	369.53921139
chr14	31529077	31529083	1-HGATAA-2-chr14	34.5	+	359.84514632
chr6	4177295	4177301	1-HGATAA-1-chr6	34.5	-	352.63599767
chr3	158461534	158461540	1-HGATAA-2-chr3	31.5	-	352.23946823
chr14	23366729	23366735	1-HGATAA-1-chr14	34.5	+	348.54592287
chr2	182170724	182170730	1-HGATAA-2-chr2	31.5	+	347.54053257
chr8	131355530	131355536	1-HGATAA-1-chr8	31.5	+	346.2938888
chr11	12222906	12222912	1-HGATAA-2-chr11	31.5	+	344.54880589
chr12	47601134	47601140	1-HGATAA-1-chr12	34.5	+	344.39714697
chr2	106002546	106002552	1-HGATAA-1-chr2	34.5	+	331.66061495
chr1	179016337	179016343	1-HGATAA-1-chr1	34.5	+	327.47086225
chr19	51161526	51161532	1-HGATAA-1-chr19	34.5	+	326.16855012
chr11	10764567	10764573	1-HGATAA-2-chr11	31.5	+	324.70144216
chr4	154001272	154001278	1-HGATAA-2-chr4	31.5	-	324.58530837
chr17	35085016	35085022	1-HGATAA-3-chr17	34.5	+	323.51680601
chr11	5297187	5297193	1-HGATAA-1-chr11	34.5	+	322.13082367
chr6	10750100	10750106	1-HGATAA-1-chr6	34.5	-	321.63525671
chr16	21548326	21548332	1-HGATAA-2-chr16	31.5	+	319.85751909
chr12	116635110	116635116	1-HGATAA-1-chr12	34.5	-	318.83571936
chr14	65510005	65510011	1-HGATAA-1-chr14	34.5	+	318.63418452
chr1	114457022	114457028	1-HGATAA-1-chr1	34.5	+	318.49413784
chr3	98302746	98302752	1-HGATAA-1-chr3	34.5	+	315.45546771
chr10	30726160	30726166	1-HGATAA-1-chr10	34.5	+	311.750006
chr3	38765774	38765780	1-HGATAA-3-chr3	31.5	+	311.39122098
chr2	7162991	7162997	1-HGATAA-2-chr2	31.5	-	308.06039247
chr11	72767188	72767194	1-HGATAA-1-chr11	34.5	-	307.06930345
chr12	122309544	122309550	1-HGATAA-1-chr12	34.5	-	302.48306998
chr1	160959833	160959839	1-HGATAA-1-chr1	34.5	-	301.71039986
chr3	46550819	46550825	1-HGATAA-1-chr3	34.5	+	298.31930536
chr6	42060095	42060101	1-HGATAA-1-chr6	34.5	+	297.00240629
chr2	69828488	69828494	1-HGATAA-1-chr2	34.5	+	296.53737507
chr10	103210225	103210231	1-HGATAA-2-chr10	31.5	-	293.99385537

* Top 50 sites with highest binding log odds are shown.

Table S3: Summary of motifs found for GATA1 CUT&RUN (based on a subset of 5000 peaks)

MOTIF_INDEX	MOTIF_SOURCE	MOTIF_ID	FACTOR	ALT_ID	WIDTH	SITES	E-VALUE
1	DREME	HGATAA	GATA1	DREME-1	6	7321	1.1e-1163
2	MEME	BBCTTATCTBH	GATA1	MEME-1	11	344	2.6e-1082
3	DREME	AGATA	GATA1	DREME-3	5	2771	6.9e-688
4	DREME	BCTTATC	GATA1	DREME-16	7	176	7.9e-676
5	DREME	CMCDCCC	KLF1	DREME-2	7	2014	5.9E-94
6	DREME	AGATTA		DREME-7	6	1064	1.1E-86
7	DREME	CTGATAKS	GATA1	DREME-12	8	250	5.2E-58
8	DREME	MGGAAR	ETS1, FLI1	DREME-4	6	2923	1.5E-41
9	DREME	CAGMWG		DREME-8	6	3901	2.7E-40
10	DREME	RTGASTCA	NFE2	DREME-6	8	433	6.3E-35
11	DREME	RCCACA	RUNX1	DREME-5	6	1706	4.5E-33
12	DREME	CHGCC		DREME-10	5	6795	3E-29
13	DREME	CTGATTRG		DREME-14	8	173	2.4E-26
14	MEME	GGATAAGCACC		MEME-50	11	4	1.30E-24
15	MEME	YTGMTATCTCYTNCTVACAG	GATA1/TAL1	MEME-12	20	20	5.80E-24
16	DREME	CTCCDCCC	KLF1	DREME-9	8	411	3E-21
17	DREME	AMACAS		DREME-11	6	2359	4.4E-16
18	DREME	CWGTSAC	PBX3/MEIS	DREME-18	7	512	4.3E-13
19	MEME	TMTATCTSTGTDCTSCTTGKC		MEME-30	21	8	1.8E-11
20	DREME	RGAAA		DREME-13	5	4901	8.5E-11
21	MEME	RCTGCCMTCTYVTGS		MEME-32	15	13	9.1E-11
22	MEME	GCCCCGCCCTC		MEME-2	11	36	3.1E-08
23	DREME	ACGT		DREME-15	4	909	5.9E-08
24	MEME	CATCWCAGCCA	GFI1B	MEME-35	11	11	6.4E-06
25	MEME	GCTRYSAGTGABAGAGAMCA	ZBTB3	MEME-20	20	10	1.7E-05
26	DREME	CCWCCTCC		DREME-17	8	154	1.9E-05
27	MEME	TWKCWRNMASCCRSCACASAG		MEME-29	21	11	3.3E-05
28	MEME	GGVCMCAGAGG		MEME-34	11	9	7.5E-05
29	MEME	TYATTCRKCTCRGSWWGRTGASTCAGRGCC		MEME-5	30	3	0.00017
30	DREME	AGGCGTGK		DREME-20	8	52	0.00052
31	MEME	CCCAGGCGTGG		MEME-33	11	2	0.0011
32	DREME	AAAAAHAA		DREME-19	8	240	0.0016
33	MEME	CAGCCCCACCC		MEME-47	11	2	0.0022
34	MEME	CGGCCCCGC		MEME-8	9	2	0.013
35	MEME	GAKGKRRTCAGASNCTGGRCTGAGWGAAG		MEME-3	29	7	0.02
36	MEME	TCCTGCTSTKG	ZIC1/2	MEME-37	11	11	0.026
37	MEME	GCWCTGCCTCC		MEME-15	11	37	0.029

Table S4: Footprint shape and symmetry analysis* (GATA1 CUT&RUN)

Motif	Ascent rate (1)	R^2 (coefficient determination)	Peak1 position	Descent rate (2)	R^2 (coefficient determination)	Peak2 position	Footprint Symmetry Score	Δrates (1-2)
DREME-16.BCTTATC	0.017885	0.952077	91	-0.015968	0.972970	129	0.032564	0.001917
DREME-1.HGATAA	0.014891	0.969814	75	-0.018961	0.940017	113	0.032266	0.004070
MEME-1.BBCTTATCTBH	0.018207	0.941735	92	-0.015034	0.972119	130	0.031761	0.003173
DREME-14.CTGATTRG	0.022813	0.982532	77	-0.007001	0.929999	114	0.028925	0.015812
MEME-50.GGATAAGCACC	0.007829	0.937589	80	-0.021961	0.967465	113	0.028587	0.014132
DREME-7.AGATTA	0.006539	0.801149	76	-0.023579	0.967788	113	0.028058	0.017040
DREME-12.CTGATAKS	0.008038	0.932221	76	-0.020616	0.984048	110	0.027780	0.012578
MEME-5.TYATTCRKCTCRGSWWGRTGASTCAGRGCC	0.006759	0.887717	75	-0.021965	0.979954	132	0.027525	0.015206
DREME-19.AAAAAHAA	0.025478	0.987155	93	-0.004194	0.515339	173	0.027312	0.021285
DREME-9.CTCCDCCC	0.024397	0.990011	90	-0.003625	0.693915	145	0.026668	0.020771
DREME-6.RTGASTCA	0.005535	0.836229	62	-0.022152	0.984747	113	0.026443	0.016618
MEME-8.CGGCCCCGC	0.023271	0.983471	93	-0.004910	0.705149	147	0.026348	0.018361
MEME-37.TCCTGCTSTKG	0.023555	0.979295	91	-0.003879	0.823788	139	0.026263	0.019676
MEME-32.RCTGCCMTCTYVTGS	0.021665	0.973954	93	-0.005579	0.898509	142	0.026114	0.016086
MEME-20.GCTRYSAGTGABAGAGAMCA	0.005315	0.874646	78	-0.021758	0.979390	122	0.025958	0.016442
MEME-33.CCCAGGCGTGG	0.005168	0.805398	59	-0.022106	0.984650	114	0.025930	0.016938
DREME-2.CMCDCCC	0.022766	0.984864	90	-0.004861	0.719514	145	0.025919	0.017905
DREME-8.CAGMWG	0.004109	0.834260	74	-0.022751	0.975606	112	0.025624	0.018642
MEME-29.TWKCWRNMASCCRSCACASAG	0.005768	0.905974	83	-0.020632	0.981285	124	0.025472	0.014864
MEME-30.TMTATCTSTGTDCTSCTTGKC	0.006531	0.922546	89	-0.019674	0.972762	122	0.025163	0.013143
MEME-3.GAKGKRRTCAGASNCTGGRCTGAGWGAAG	0.004464	0.791282	95	-0.021919	0.984111	132	0.025103	0.017455
MEME-15.GCWCTGCCTCC	0.003754	0.740657	65	-0.024095	0.913687	112	0.024796	0.020341
DREME-17.CCWCCTCC	0.024659	0.990057	88	-0.001191	0.181527	110	0.024630	0.023469
MEME-47.CAGCCCCACCC	0.023026	0.985668	93	-0.002392	0.518524	120	0.023936	0.020634
DREME-10.CHGCC	0.004036	0.700540	70	-0.021109	0.982761	109	0.023572	0.017073
MEME-35.CATCWCAGCCA	0.003658	0.715174	78	-0.021315	0.982304	112	0.023554	0.017657
MEME-2.GCCCCGCCCTC	0.022955	0.988390	95	-0.002275	0.327758	110	0.023434	0.020680
DREME-11.AMACAS	0.004115	0.711195	55	-0.021011	0.974483	111	0.023402	0.016897
DREME-18.CWGTSAC	0.004215	0.750771	64	-0.020574	0.963616	109	0.022989	0.016359
DREME-5.RCCACA	0.003010	0.662202	79	-0.020842	0.982252	110	0.022465	0.017831
DREME-20.AGGCGTGK	0.020585	0.978716	96	-0.003452	0.656984	126	0.022415	0.017133
DREME-4.MGGAAR	0.001027	0.116794	96	-0.020259	0.967402	113	0.019719	0.019232
MEME-34.GGVCMCAGAGG	0.001975	0.280822	96	-0.019529	0.955259	114	0.019210	0.017554

* For each footprint, we fit the data with A exp(B * x). Ascent rate refers to the parameter B estimated on the left arm of the footprint. Descent rate refers to the parameter B estimated on the right arm of the footprint. See **Fig 3**.
