# Supplementary material for: CUT&RUNTools: a flexible pipeline for CUT&RUN processing and footprint analysis
Source: Genome Biol. 2019 Sep 9;20:192. doi: 10.1186/s13059-019-1802-4 (PMC6734249; doi:10.1186/s13059-019-1802-4)
Supplement: Supplementary file 2 — Supplementary Figures S1 - S11. (PDF 4187 kb) [file 13059_2019_1802_MOESM2_ESM.pdf]

Figures S1-S11

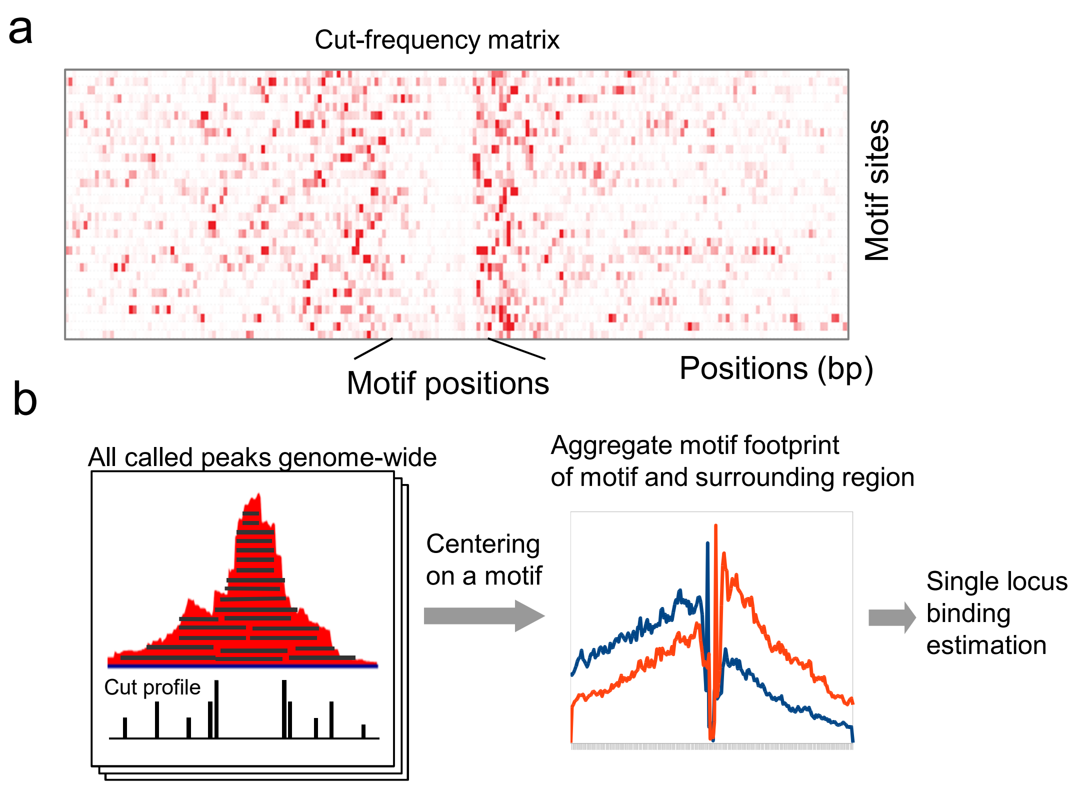

Fig S1: a. Illustrative example of a cut frequency matrix. Rows: motif sites. Columns: (-100bp, motif, +100bp) region. Cuts are tabulated at each position of the motif site. Darkness of red indicates more cuts. b. Steps to construct an aggregate motif footprint. First, all peaks are scanned for motif sequence. Then, for each motif occurrence, we obtain cut frequency on (-100bp, +100bp) region centered on motif. Next, CENTIPEDE computes the posterior probability of cutting at each position of the motif site using an iterative procedure based on the spatial distribution of reads around motif. The output is the posterior probability of cutting per position, a number weighted from all motif sites according to each site's overall probability value.

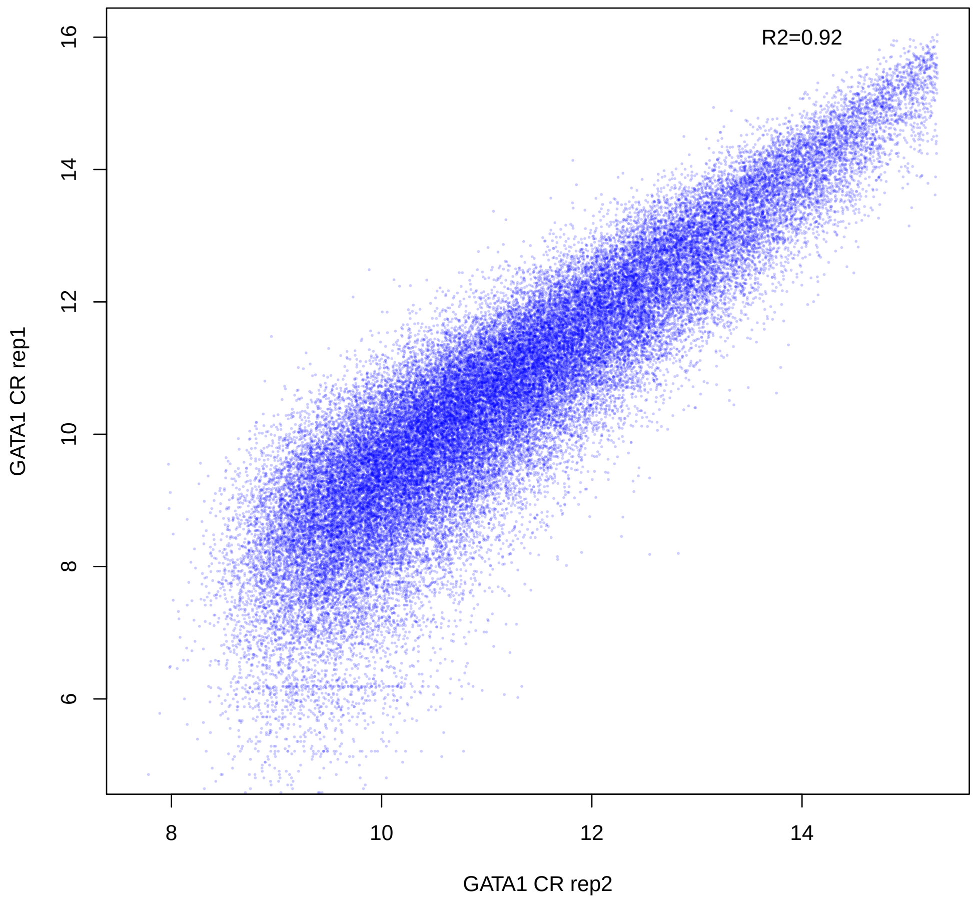

Fig S2: Correlation between GATA1 CUT&RUN replicates. Peak signal (i.e. fragment coverage) was computed for each replicate 1 and 2 for 40,000 shared peaks between the replicates.

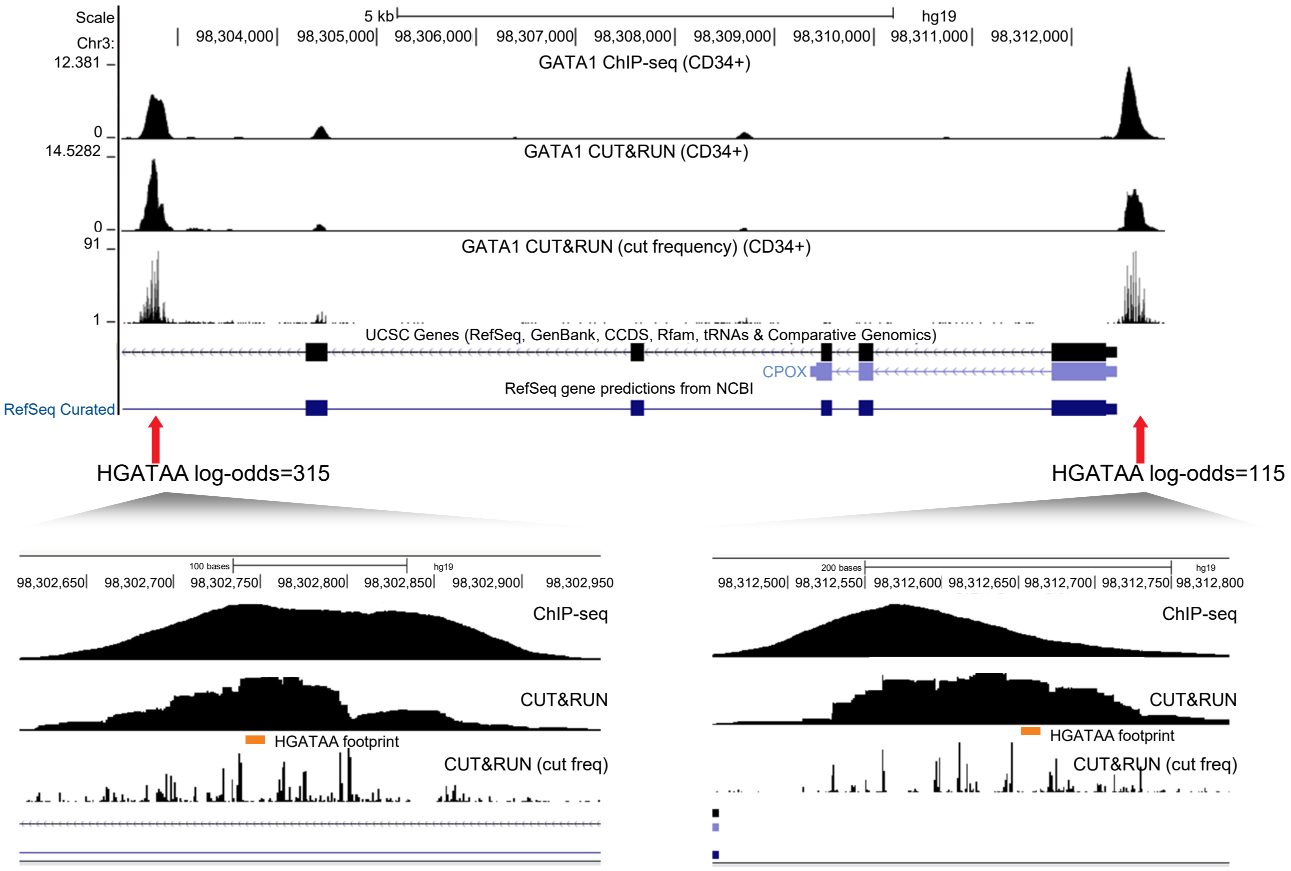

Fig S3: Validation of two GATA1 sites at the CPOX gene locus. GATA1 binding at these sites is confirmed based on a previous report [35]. CUT&RUNTools predicts binding with highly significant log odds of 315 and 115 respectively, which are ranked in the top 0.1% and 2% of all sites. A zoom-in view is provided on each site to show 1) ChIP-seq profile, 2) CUT&RUN coverage profile, 3) CUT&RUN cut frequency profile. Cut frequency profile shows a series of footprints that may correspond to GATA1 and other transcription factors.

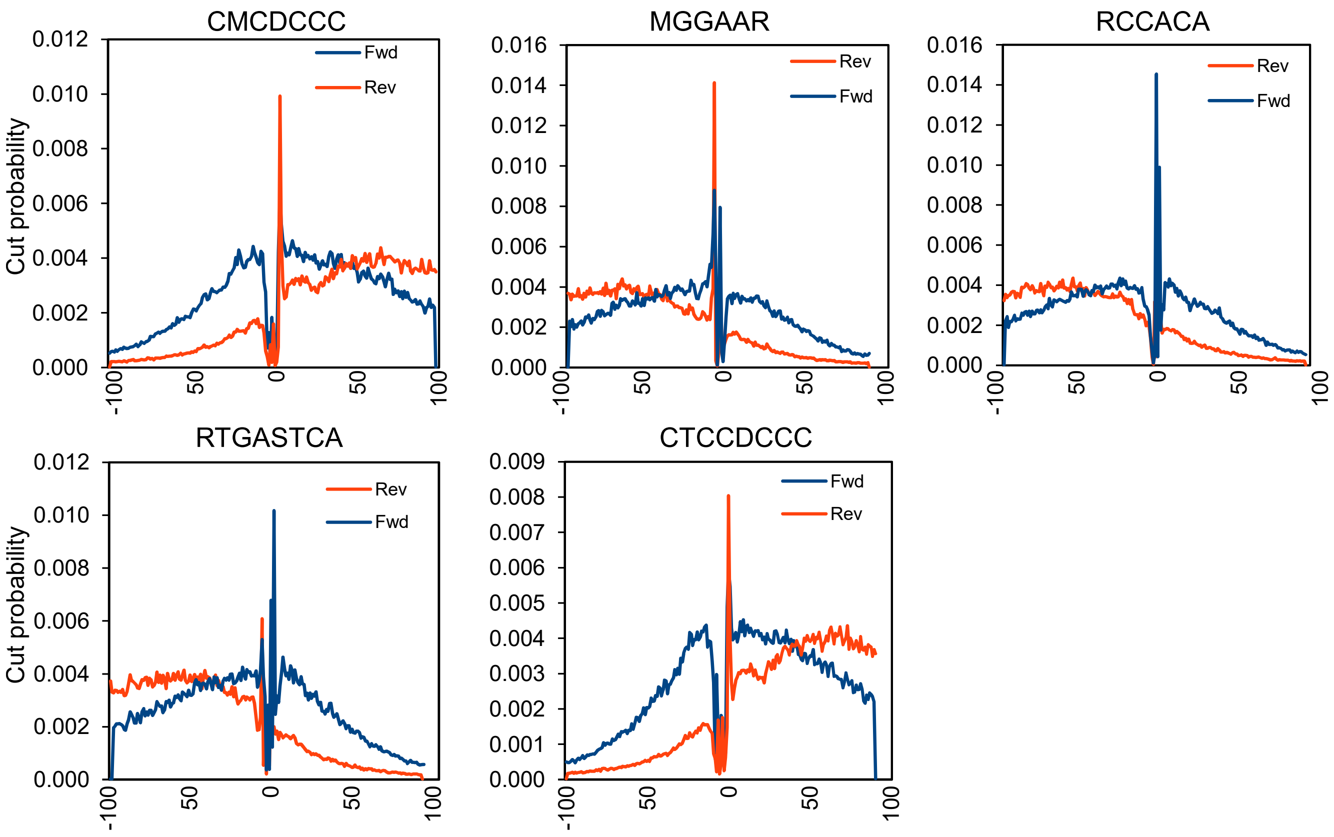

Fig S4: Examples of secondary motifs detected from GATA1 CUT&RUN experiment. Secondary motifs in general have asymmetrical footprint shapes and usually indicate factors that co-occur with the primary antibody. From left to right, these motifs correspond to factors KLF1, ETS1, RUNX1, NFE2, and KLF1.

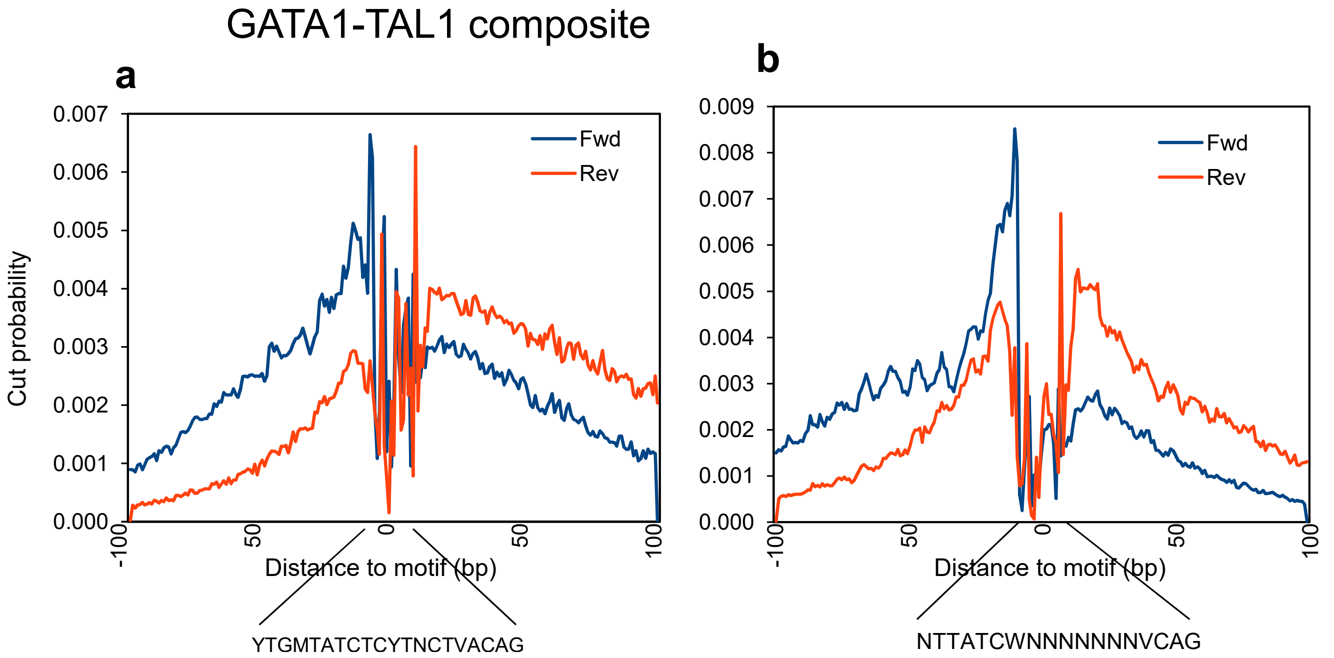

Fig S5: GATA1-TAL1 composite motif footprint a. using the motif that is found *de novo*, and b. using the motif that is from JASPAR motif database.

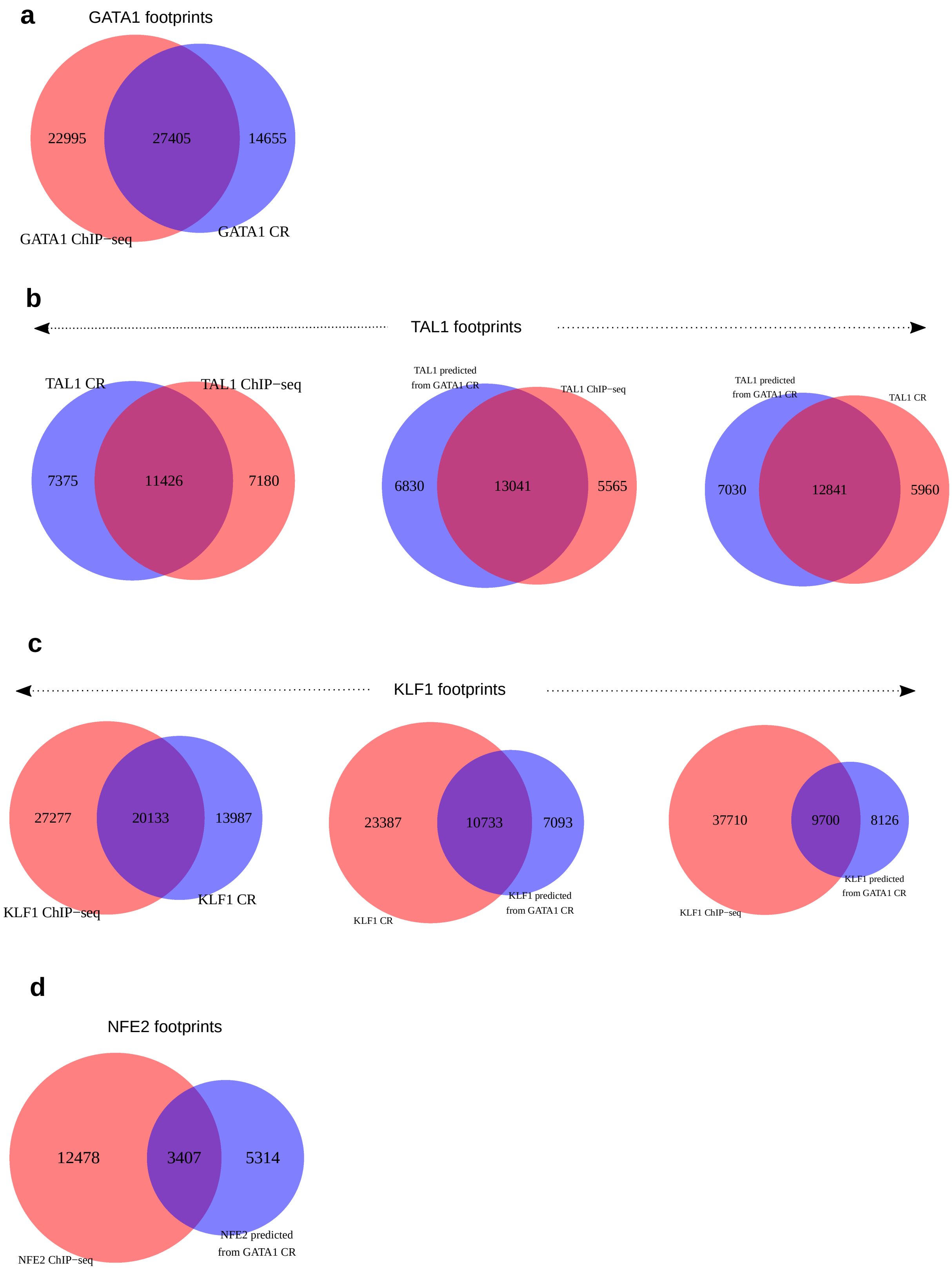
a
GATA1 footprints
22995
27405
14655
GATA1 ChIP−seq
GATA1 CR
b
TAL1 footprints
TAL1 CR
TAL1 ChIP−seq
7375
11426
7180
TAL1 predicted
from GATA1 CR
TAL1 ChIP−seq
6830
13041
5565
TAL1 predicted
from GATA1 CR
TAL1 CR
7030
12841
5960
c
KLF1 footprints
27277
20133
13987
KLF1 ChIP−seq
KLF1 CR
23387
10733
7093
KLF1 predicted
from GATA1 CR
KLF1 CR
37710
9700
8126
KLF1 predicted
from GATA1 CR
KLF1 ChIP−seq
d
NFE2 footprints
12478
3407
5314
NFE2 predicted
from GATA1 CR
NFE2 ChIP−seq

Fig S6: GATA1 co-factor analyses. a. GATA1 ChIP-seq vs. GATA1 CUT&RUN comparison. Number of overlapping GATA1 footprints (i.e. GATA1 motif sites located in overlapped peaks) is shown. b. TAL1 footprints. *Left*: comparison between ChIP-seq and CUT&RUN. *Middle*: number of TAL1 sites in GATA1 CUT&RUN that overlap with TAL1 ChIP-seq. *Right*: number of TAL1 sites in GATA1 CUT&RUN that overlap with TAL1 CUT&RUN. c. KLF1 footprints. Same as b, except that the transcription factor is KLF1. d. NFE2 ChIP-seq vs. NFE2 predicted from GATA1 CUT&RUN.

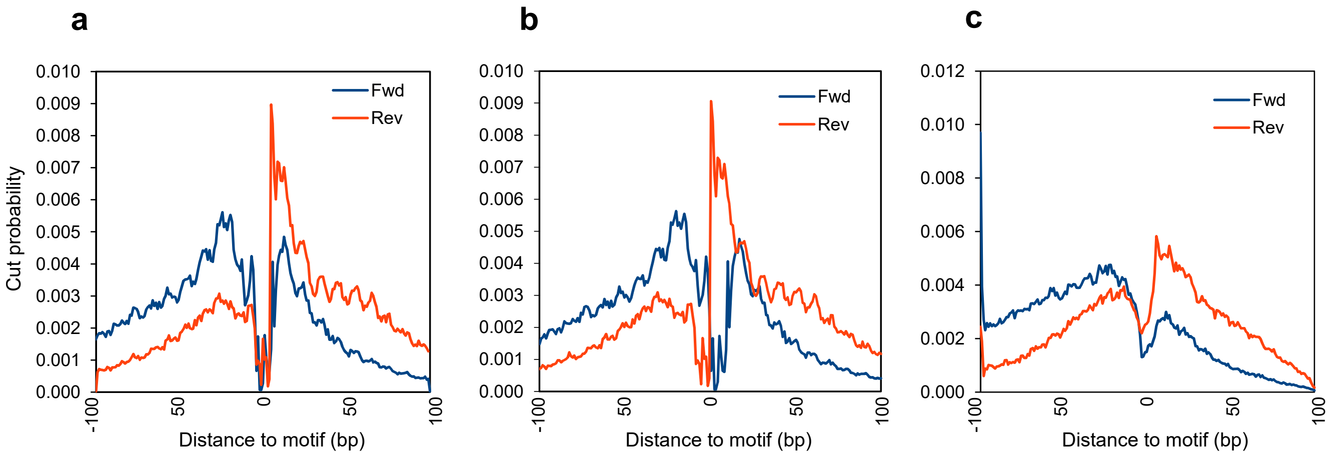

Fig S7: Comparisons of HGATAA motif footprint using the cut matrix enumerated by a. CUT&RUNTools, b. Atactk, and c. CENTIPEDE.tutorial. Options b. and c. are unsuitable for CUT&RUN data as they are specifically designed for ATAC-seq and DNase-seq data respectively. They miscalculated the cut matrix. See Fig S8 for numerical comparisons of the cut matrices.

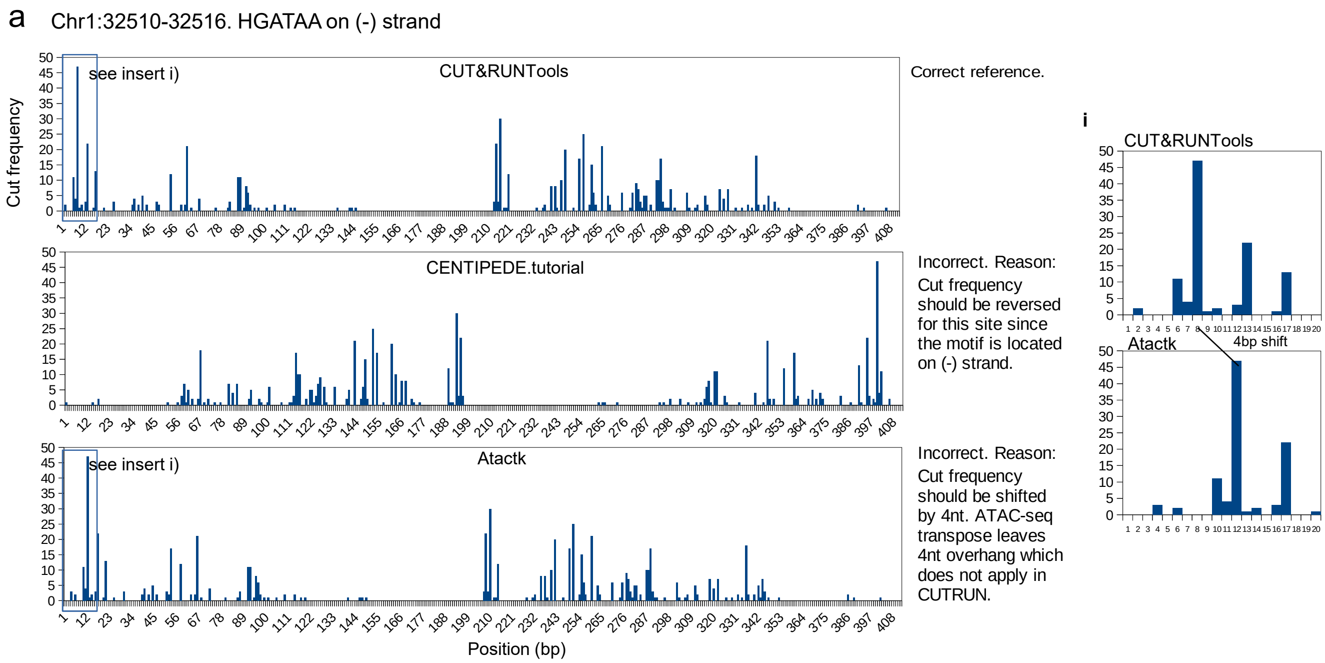

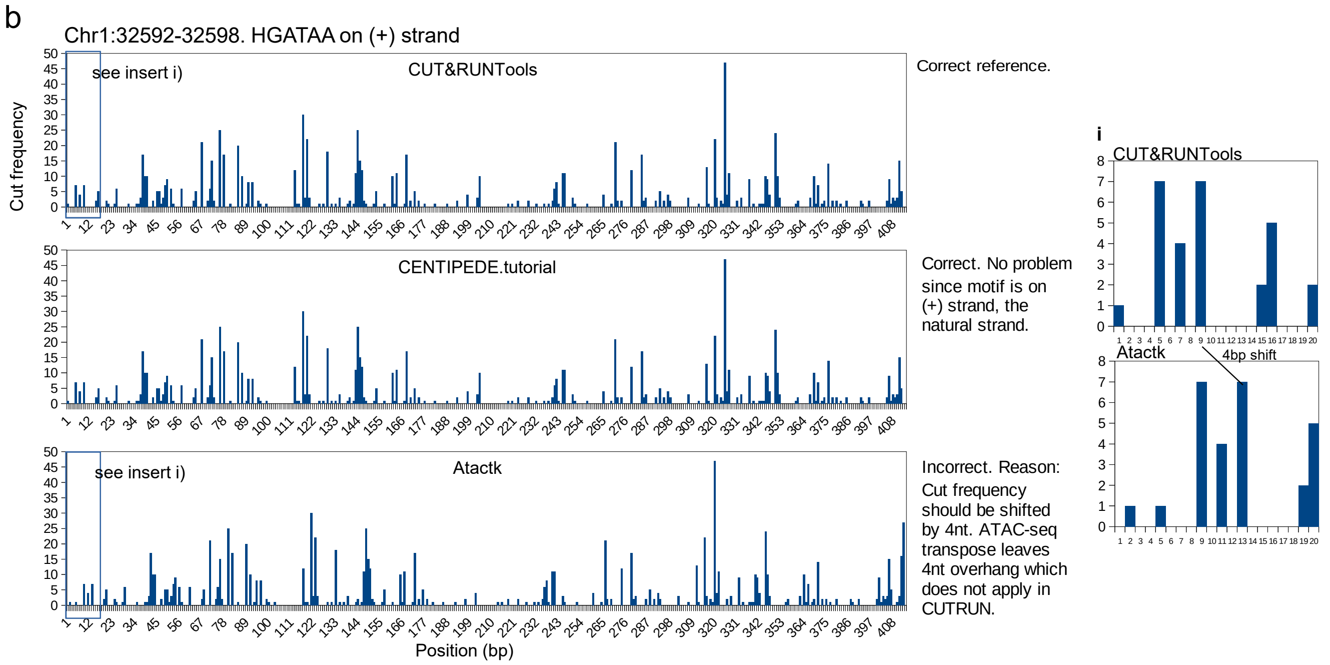

Fig S8: a. Cut matrix comparison between CUT&RUNTools, CENTIPEDE.tutorial, and Atactk. Cut frequency is presented as a bar plot on HGATAA motif site that is located at **chr1:32510-32516, strand (-)**. Plot shows cuts in 206bp regions surrounding this site. The first 206bp indicates cuts on forward strand, and the next 206bp shows reverse strand. Insert (i) shows the zoom-in of first 20bp of CUT&RUNTools and Atactk, showing a 4bp shift between their profiles. CENTIPEDE.tutorial and Atactk both miscalculated cuts when applied to CUT&RUN data. Right-hand side shows the reason for the errors. Specifically, the cut matrix generated by CENTIPEDE.tutorial should be reversed since motif is on (-) strand, and Atactk's matrix is off by 4bp. b. Cut matrix comparison between CUT&RUNTools, CENTIPEDE.tutorial, Atactk.

Cut frequency is shown for HGATAA motif located at **chr1:32592-32598, strand (+)**. Plot shows cuts in two 206bp regions surrounding motif (-100bp, motif, +100bp). First 206bp: forward strand. Next 206bp: reverse strand. Insert (i) shows the zoom-in of first 20bp of CUT&RUNTools and Atactk. Atactk contains errors in the estimated cuts. Right-hand side shows the reason for error.

Anatomy of DNA fragment

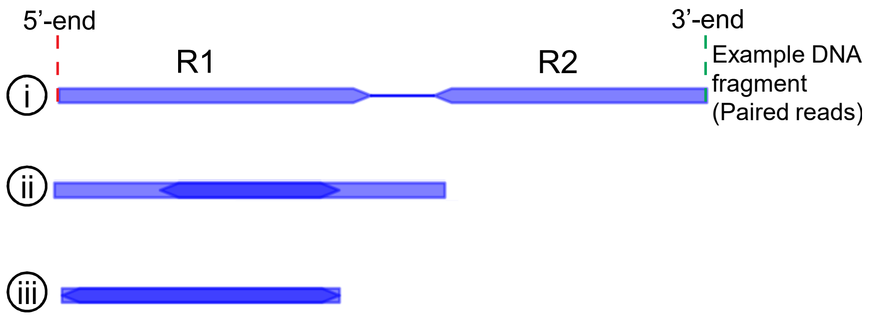

Fig S9: Anatomy of DNA fragment with both mates of a pair (R1, R2) indicated. Three scenarios of a DNA fragment could occur: i) R1 and R2 has a gap (unsequenced region) in the middle. ii) R1 and R2 overlap partially. iii) R1 and R2 completely overlap. Ends of a read do not always correspond to a fragment end, due to unsequenced region. The 5’-end of a read is used to indicate fragment ends.

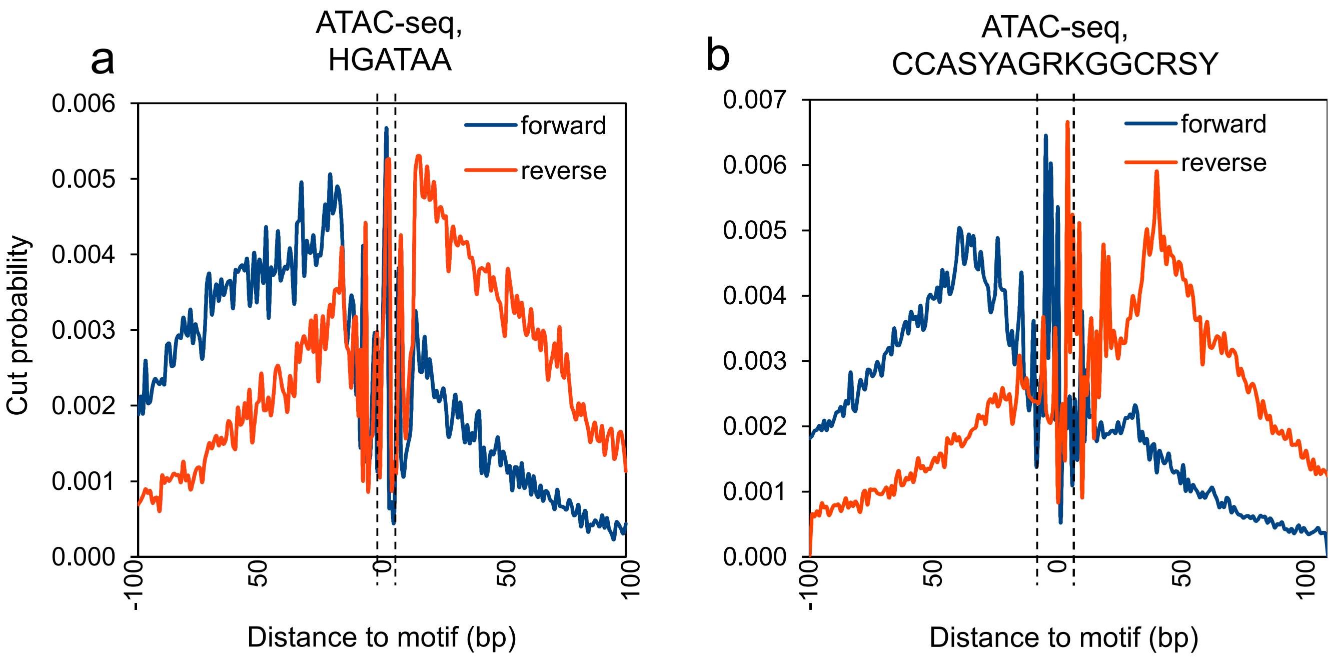

Fig S10: Application of CUT&RUNTools on ATAC-seq data. Motif footprinting analysis was performed on two given motifs, GATA1, and CTCF on ATAC-seq HUDEP-2 cells. CUT&RUNTools was able to generate a motif footprint in each case. A cut site offset of 4bp was used.

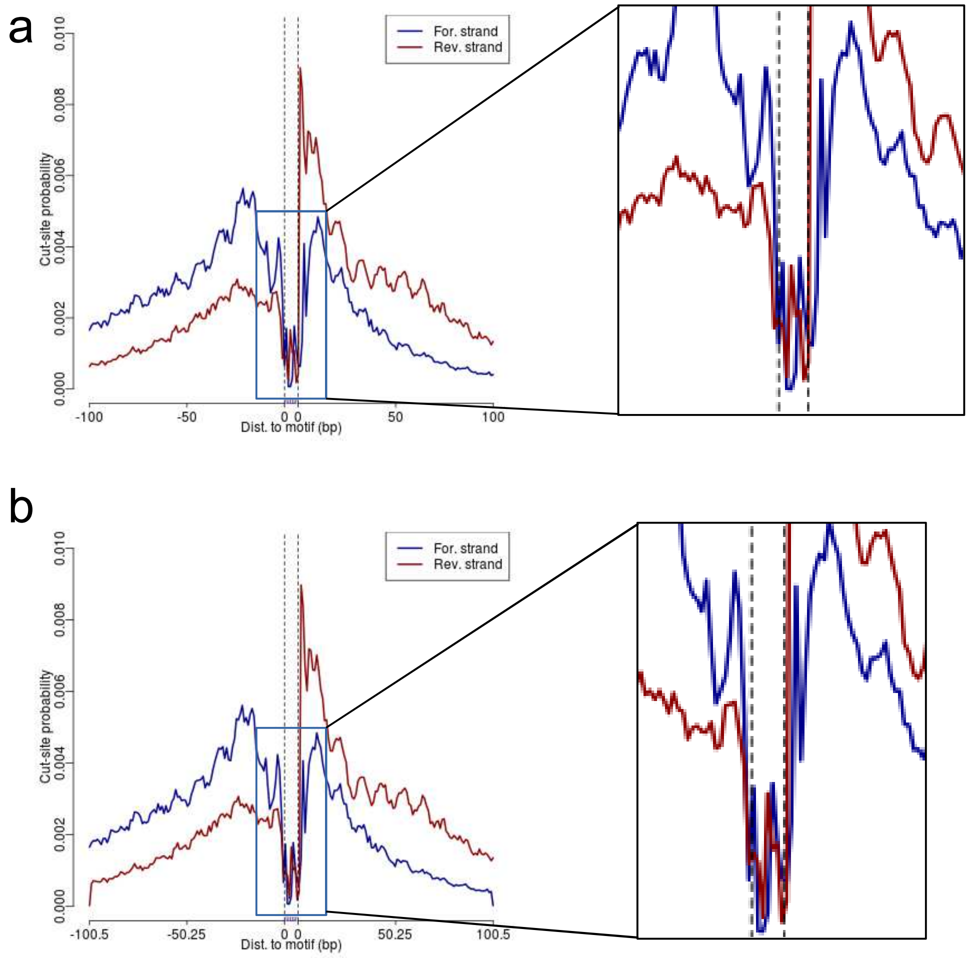

Fig S11: a. With cut site offset set to 0, atactk-generated cut matrix creates a motif footprint that is close to the correct solution (see b), but is still incorrect because the forward and reverse strands are misaligned by 1bp (see insert). b. The correct motif footprinting plot estimated by CUT&RUNTools.
