# Supplementary material for: CUT&RUNTools: a flexible pipeline for CUT&RUN processing and footprint analysis
Source: Genome Biol. 2019 Sep 9;20:192. doi: 10.1186/s13059-019-1802-4 (PMC6734249; doi:10.1186/s13059-019-1802-4)
Supplement: Supplementary file 3 — Review history. (DOCX 16 kb) [file 13059_2019_1802_MOESM3_ESM.docx]

Review History

**First round of review**

**Reviewer 1**

**Were you able to assess all statistics in the manuscript, including the appropriateness of statistical tests used?**

Yes, and I have assessed the statistics in my report.

**Have you been able to install and test the software?**

No

**Comments to author:**

CUT&RUN is quickly replacing ChIP-seq as an experimental workhorse for determining transcription factor binding. The authors propose a novel analysis pipeline composed of existing tools that provides a new and improved way of analyzing this type of data. The individual tools are not new, but their application to this type of data is novel. The authors also show that using the footprint pattern left by the cleavage of the pA-MN fusion protein to the antibody tagged DNA:protein interactions can give more information in terms of the exact binding locations and interactions with other co-binding proteins. The method is well described but the authors could further highlight in which cases it will provide additional information to just doing a peak analysis. I think the GATA1 example is a good start but more examples of known binding partners leaving different footprints for different factors would be a good addition to further increase the profile of the manuscript and the method. In any case, I think the method will be useful to any biologist interested in gene regulation and producing CUT&RUN or ATAC-seq data as well.

Major comments:

1) It may be good to test the algorithm for other factors other than GATA1. If some of the co-factors Cut&Run data was also available, it may be interesting to compare.

2) For the software package it may also be good to provide an option that can start from known motif databases such as JASPAR so the match finding tool can use known motifs as well.

Minor comments:

1) The quality of some of the figures should be improved, some look like screenshots.

2) One of the references ([15]) seems mis-formatted. For sake of consistency I would also mention CENTIPEDE in page 3 line 56 together with [15], as it is done for MACS[11] and MEME[12] in the same page line 25. If some of these tools could be replaced by another method (e.g. the aligners) keeping just at the methods would be fine.

3) Parts of the caveats that are in the Quality Control Metrics should probably be mentioned at the main part of the paper. Depending of the type of downstream analysis, for example comparing across different species, differences in mappability or sequence composition maybe an issue. I think this should also be mentioned.

**Reviewer 2**

**Were you able to assess all statistics in the manuscript, including the appropriateness of statistical tests used?**

Yes, and I have assessed the statistics in my report.

**Have you been able to install and test the software??**

Yes

**Comments to author:**

In this study, the authors developed a flexible, general pipeline, CUT&RUNTools for facilitating the identification of chromatin-associated protein binding and genomic footprinting analysis from

antibody-targeted CUT&RUN primary cleavage data. CUT&RUNTools extracts endonuclease cut site information from sequences of short read fragments and produces single-locus binding estimates, aggregate motif footprints, and informative visualizations to support the high resolution mapping capability of CUT&RUN.

1. It needs to specify how to optimize the statistical cutoff values for each step in the CUT&RUNTools tool.

2. In GATA1 data, it is unclear if the authors perform the biological replicates. If not, they need to do it.

3. In GATA1 data, please provide the details of direct binding vs indirect (tethering) binding sites, and validate them using co-IPs and other types of experimental techniques.

4. It needs to be tested more data sets to demonstrate the efficiency and broad applicability of the tool. For example, FOXA1 and GATA2 are two typical pioneer factors. 50-70% of these factors usually bind to nucleosome in some cancer cells. How the CUT&RUN vs ChIP-seq vs ChIP-exo of these factors would be different in terms of binding patterns?

Please consider the following questions:

-Is the Software likely to be of broad utility? Is it easy to install and use?

Not sure, it needs to be tested on more data sets.

-Does the software represent a significant advance over previously published tools (as demonstrated by direct comparison with available related software)?

Not sure. it needs to be tested on more data sets.

**Authors Response**

**Point-by-point responses to the reviewers’ comments:**

Reviewer #1:

CUT&RUN is quickly replacing ChIP-seq as an experimental workhorse for determining transcription factor binding. The authors propose a novel analysis pipeline composed of existing tools that provides a new and improved way of analyzing this type of data. The individual tools are not new, but their application to this type of data is novel. The authors also show that using the footprint pattern left by the cleavage of the pA-MN fusion protein to the antibody tagged DNA:protein interactions can give more information in terms of the exact binding locations and interactions with other co-binding proteins. The method is well described but the authors could further highlight in which cases it will provide additional information to just doing a peak analysis. I think the GATA1 example is a good start but more examples of known binding partners leaving different footprints for different factors would be a good addition to further increase the profile of the manuscript and the method. In any case, I think the method will be useful to any biologist interested in gene regulation and producing CUT&RUN or ATAC-seq data as well.

*We thank the reviewer for these positive comments. We agree that the tool is of broad interest to biologists doing not just CUT&RUN but ATAC-seq as well.*

Major comments:

1) It may be good to test the algorithm for other factors other than GATA1. If some of the co-factors Cut&Run data was also available, it may be interesting to compare.

*We have also tested the tool with two other factors: TAL1and KLF1 – both are known to be important regulators in erythroid cells. Our analysis showed that such data can be used to examine cofactor relationship with GATA1.*

*Specifically, we have found 19,871 TAL1 sites located in GATA1 CR peaks. We found that 12,841 of these are actual TAL1 sites verified by TAL1 CR experiment. This corresponds to an overlap of 64.6%. When compared with TAL1 ChIP-seq experiment, 13,041 of 19,871 (65.6%) TAL1 sites overlap with TAL1 peaks in TAL1 ChIP-seq. Overall, GATA1 CR successfully located a large fraction of co-occupying TAL1 sites.*

*Similarly, KLF1 is another cofactor enriched among GATA1 CR peaks. Altogether, 17,826 KLF1 footprints are found in GATA1 CR. 9,700 of 17,826 (54.4%) are confirmed to have peaks in KLF1 ChIP-seq. 10,733 of 17,826 (60.2%) are confirmed to have peaks in KLF1 CR.*

*All of the above analyses are summarized in the new Supplementary Fig 12 that we added to the paper. CUT&RUN is thus quite informative of predicting cofactor binding locations.*

*We have also applied our tools to MYC and MAX CUT&RUN studies from Steven Henikoff’s lab. The exact details of the comparison is found in Supplementary Fig 8 and in main text. Briefly, CUT&RUN and ChIP-seq share a high degree of overlap both in terms of shared peaks and in shared motif sites in peak regions (66.9% overlap for MAX and 58% for MYC). We found a large number of co-occupied MYC and MAX sites.*

2) For the software package it may also be good to provide an option that can start from known motif databases such as JASPAR so the match finding tool can use known motifs as well.

*We thank the reviewer for the valuable suggestion. In this revision, we have added an option to allow users easily test motif footprints for known motifs such as from the JASPAR database that you mentioned. To do so, users can simply download the motif from the JASPAR website in the *.meme format (for example GATA1 motif, go to http://jaspar.genereg.net/matrix/MA0035.3/?revcomp=1, click on MEME button). With the downloaded motif, run the new provided script in CUT&RUNTools, called*

*python3 generate.footprinting.factor.specific.centipede.py -b MA0035.2.GATA1.meme -p 0.001 -n GATA1 -o fimo.GATA1.result*

*where the options mean:*

*-b Designated motif in MEME format*

*-p P-value for FIMO motif scanning*

*-n Name of factor*

*-o Output directory*

*This Python script will generate a BASH script called integrate.footprinting.GATA1.centipede.sh, which can be run on a CUT&RUN experiment narrowPeak file (generated by MACS2). For example:*

*./integrate.footprinting.GATA1.centipede.sh GATA1_D9_30min_S12_aligned_reads_peaks.narrowPeak*

*The output files will be located in the fimo.GATA1.result directory:*

*└---- fimo.GATA1.result*

*└--- GATA1_D9_30min_S12_aligned_reads_peaks*

*└--- fimo2.MA0035.2.GATA1*

*└--- cisml.xml*

*└--- fimo.bed*

*└--- fimo.cuts.freq.txt*

*└--- fimo.gff*

*└--- fimo.html*

*└--- fimo.lambda.txt*

*└--- fimo.logratio.bed*

*└--- fimo.logratio.txt*

*└--- fimo.png*

*└--- fimo.postpr.txt*

*└--- fimo.txt*

*└--- fimo.xml*

*The output files are fimo.bed, fimo.cuts.freq.txt, and fimo.logratio.txt, which contain information about the binding site locations, cut frequency matrix, and binding log odds for the provided GATA1 motif.*

Minor comments:

1) The quality of some of the figures should be improved, some look like screenshots.

*We thank the reviewer for pointing out this issue. In the revision, we have regenerated a number of main and supplementary figures as high-quality SVG/PDF format, including Figure 1 and Supplementary Figures 2, 4-7. We have also changed the presentation layout of Figures 1 and 2 to improve clarity.*

2) One of the references ([15]) seems mis-formatted. For sake of consistency I would also mention CENTIPEDE in page 3 line 56 together with [15], as it is done for MACS[11] and MEME[12] in the same page line 25. If some of these tools could be replaced by another method (e.g. the aligners) keeping just at the methods would be fine.

*We have corrected the format of ref [15] and reworded the corresponding text as follows:*

*“Then, CUT&RUNTools estimates a probabilistic bimodal clustering model derived from the CENTIPEDE package [15], and assigns a binding probability score, expressed as log-odds, to each motif occurrence based on the model.”*

3) Parts of the caveats that are in the Quality Control Metrics should probably be mentioned at the main part of the paper. Depending of the type of downstream analysis, for example comparing across different species, differences in mappability or sequence composition maybe an issue. I think this should also be mentioned.

*We thank the reviewer for this nice suggestion. We have added the following discussion in the main text about the caveats of CUT&RUNTools, For example, the problem of short fragments generating a lot of duplicate fragments, and the sequence complexity of the bound DNA fragments are two issues.*

*We should note that some of the mappability or sequence composition issues are caused by the antibody and TFs, and not specific to the CUT&RUN technology. As such, the issues will be encountered no matter what technology is used.*

*We added the following text to the main text:*

*“Quality control measures such as alignment rate, fragment duplication rate may be used, but we note that due to differences in mappability and sequence composition of the antibody bound DNA, some factors will inherently have a low complexity of the binding regions and an increased fragment duplication rate. Users thus need to make judgement calls (for example whether or not to remove duplicates) on a factor-dependent basis. We advice an interpretation that is aided by motifs and replicate analysis.”*

Reviewer #2:

In this study, the authors developed a flexible, general pipeline, CUT&RUNTools for facilitating the identification of chromatin-associated protein binding and genomic footprinting analysis from antibody-targeted CUT&RUN primary cleavage data. CUT&RUNTools extracts endonuclease cut site information from sequences of short read fragments and produces single-locus binding estimates, aggregate motif footprints, and informative visualizations to support the high resolution mapping capability of CUT&RUN.

*We thank the reviewer for recognizing the numerous strengths of CUT&RUNTools..*

1. It needs to specify how to optimize the statistical cutoff values for each step in the CUT&RUNTools tool.

*There are indeed statistical cutoff values associated with some of steps of the pipeline. These are typically associated with the level of confidence the user wants. Some of the cutoffs (such as P-values) are associated with the individual components of the pipeline, such as MACS2, MEME, and we feel that users are better to look up the manual of these tools for how to set these values.*

*However, we understand that there are a few critical parameters that can determine the success of using CUT&RUNTools, so we provide some guidelines about how to best set the cutoff values for these parameters. We make clear that parameters are by no means optimal. They are guidelines to arrive at reasonable values.*

*1)	P-value cutoff for MACS2. This determines the number of peaks called by MACS2. MACS2 by default will use a Q-value (minimum FDR) of 0.01 in place of P-value. This is also the default value in CUT&RUNTools and is satisifactory for most cases. Though when comparing CR of different sequencing depth, we increase stringency of P-value of the higher-depth experiment to make the number of peaks comparable to the lower-depth experiment (as an additional filter to Q-value).*

*2)	Use of replicate to aid the setting of P-value. We always recommend doing replicates. With two replicates, we can then gauge at the reproducibility of peaks and motifs. One reasonable way to set peak calling cutoff (Pvalue) is to find a cutoff/plateau point that maximizes the overlap of peaks, or the correlation of peak signals remains high across replicates.*

*3)	Motif scanning Pvalue. We set this to the recommended value determined by FIMO as part of MEME suite. We may need to relax the default cutoff if FIMO does not pick up the motif instance in the sequence with the provided PWM. Then with all motifs located in peak sequences, we can calculate the fraction of peaks containing motif at every Pvalue cutoff. We expect to find >20-50% of peaks should contain the motif.*

*4)	Logodds cutoff for calling TF binding sites. Logodds have an interpretable meaning as it is associated with probability: log(p/(1-p)). So a log odds cutoff of 2.9 means Prob=0.95. We recommend setting this in the 2 to 5 range (default is 3). Log odds are comparable across experiments only if reads are scaled to the same sequencing depth.*

2. In GATA1 data, it is unclear if the authors perform the biological replicates. If not, they need to do it.

*We have generated another GATA1 replicate, and included it in this paper.*

*We found a large overlap of peaks and high correlation of signal strength in the second replicate of GATA1 CUT&RUN comparing with the GATA1 experiment in this paper. Peak overlap is over 40,000 and correlation of replicates is R2=0.92 (see scatterplot in Supplementary Figure 11).*

3. In GATA1 data, please provide the details of direct binding vs indirect (tethering) binding sites, and validate them using co-IPs and other types of experimental techniques.

*We thank the reviewer for this suggestion. In our study, direct binding means the presence of primary motif in the peak and has a positive log odds score. All othe peaks are considered indirect, and could be due to cofactor binding or 3D chromatin interactions.*

*To validate the co-factors, KLF1, NFE2, and TAL1 are well known co-factors of GATA1. The fact that GATA1 CR picked up KLF1, NFE2, and TAL1 motifs indicated to us that it might be useful for finding cofactor binding sites.*

*We have indeed compared and validated the KLF1, NFE2, and TAL1 motifs within the GATA1 CR by using ChIP-seq and CUT&RUN experiments of these cofactors. New CUT&RUN experiments have been performed by us. See Supplementary Figure 12 for details. To reiterate the response to Reviewer 1, in the GATA1 CUT&RUN (shortened CR) data, out of 19,871 TAL1 sites located in GATA1 CR peaks. 64.6% are actually TAL1 sites in TAL1 CR experiment; 65.6% overlap with TAL1 peaks in TAL1 ChIP-seq. For KLF1, we found similar high proportion (54%) of KLF1 sites predicted from GATA1 CR to have evidence from KLF1 ChIP-seq. Full details of the analysis are discussed in the main text and shown in Supplementary Fig 12 that we added to the paper.*

4. It needs to be tested more data sets to demonstrate the efficiency and broad applicability of the tool. For example, FOXA1 and GATA2 are two typical pioneer factors. 50-70% of these factors usually bind to nucleosome in some cancer cells. How the CUT&RUN vs ChIP-seq vs ChIP-exo of these factors would be different in terms of binding patterns?

*We thank the reviewer for this suggestion. To illustrate a broad applicability of our tool, we have now compared CUT&RUN with ChIP-seq datasets that have been generated for several factors from other labs. For example, we have compared the CUT&RUN data for Max, Myc that were done in the original Steven Henikoff’s paper, with the corresponding ChIP-seq experiments from K562 cells in the ENCODE database. Unfortunately, we are not familiar with the biology FOXA1 in breast cancer cells, but we hope that the Max example would serve to illustrate the applicability equally well.*

*Analyses of Max CR and Myc CR using CUT&RUNTools identified 23,153 and 6,996 peaks respectively, and recovered the respective TF motifs as primary. See Supplementary Figure 13. As expected, CR compares favorably with ChIP-seq (see Venn diagrams in SFig 13d). It has improved resolution over ChIP-seq (SFig 13a, e). Full details are located in the main text. Not only do we discover a motif footprint, interestingly, Max CR revealed a 10-bp periodic cut pattern in its summary footprint profile (SFig 13b), which is supported by the Max binding to the nucleosomal DNA. This observation is supported by DNase I footprinting of Max (https://mcb.asm.org/content/mcb/14/6/4097.full.pdf) where there’s reported enhanced cleavage in nucleosomal DNA separated by areas of increased protection (at ~10bp periodicity). Like Max, we believe that CUT&RUN is very useful for studying the binding of transcription factors to nucleosomal DNA.*

*We have also added our own data (TAL1 CUT&RUN, and KLF1 CUT&RUN, which each has its own corresponding ChIP-seq from the public database).*

*About ChIP-exo: CUT&RUNTools is applicable to any paired-end sequencing data derived from endo/exo-nuclease digestion. As such it is in principle also applicable to ChIP-exo. Steven Henikoff’s original CUT&RUN paper has compared CUT&RUN to ChIP-exo and found it to have superior resolution to ChIP-exo. Since our paper is a pipeline development paper, we feel that it is not our goal to repeat experiments of the original CUT&RUN paper.*

*The main conclusion from our already existing GATA1 ChIP-seq vs CUT&RUN comparison (and several others included in this revision TAL1, MAX, MYC) is that ChIP-seq data can only identify a peak at binding sites, but CUT&RUN can also identify clear footprint in the middle. This is of course in addition to the improved resolution of CUT&RUN over ChIP-seq.*

**Second round of review**

**Reviewer 1**

The authors carefully addressed all my major points and I don't have any further comments except perhaps moving some of the supplementary figures as additional panes of the main figures.

**Reviewer 2**

The authors have addressed my concerns.

**Authors Response**

**Point-by-point responses to the reviewers’ comments:**

We thank the reviewers for their positive comments. As suggested, we have moved two supplementary figures to the main figures.
